# Supplementary material for: Maternal and environmental Impact assessment on Neurodevelopment in Early childhood years (MINE): a prospective cohort study protocol from a low, middle-income country
Source: BMJ Open. 2023 Jul 9;13(7):e070283. doi: 10.1136/bmjopen-2022-070283 (PMC10335476; doi:10.1136/bmjopen-2022-070283)
Supplement: Supplementary data [file bmjopen-2022-070283supp001.pdf]

Supplementary Information

Table 1: Parameters for FLAIR and SWI sequences

Sequence	FLAIR	FSBB SWI (1)	FSBB SWI (2)
Orientation	Coronal	Axial	Axial
TR (ms)	10000	29	29
TE (ms)	120	20	20
FA (degrees)	90	10	10
FOV (mm)	200x200	220x220	220x220
Matrix size	640x640	576x576	576x576
#Slices	24	9	104
Thickness (mm)	4	20	2
Spacing (mm)	5	1	1
Voxel size (mm^3)	0.312x 0.312x5	0.382x 0.382x10	0.382x 0.382x1
Phase Encoding Direction	i; R to L	j; A to P	j; A to P
Time (s)	02:20	02:10	02:10
