# Supplementary material for: Maternal and environmental Impact assessment on Neurodevelopment in Early childhood years (MINE): a prospective cohort study protocol from a low, middle-income country
Source: BMJ Open. 2023 Jul 9;13(7):e070283. doi: 10.1136/bmjopen-2022-070283 (PMC10335476; doi:10.1136/bmjopen-2022-070283)
Supplement: Author's manuscript [file bmjopen-2022-070283.draft_revisions.pdf]

BMJ Open is committed to open peer review. As part of this commitment we make the peer review history of every article we publish publicly available.

When an article is published we post the peer reviewers' comments and the authors' responses online. We also post the versions of the paper that were used during peer review. These are the versions that the peer review comments apply to.

The versions of the paper that follow are the versions that were submitted during the peer review process. They are not the versions of record or the final published versions. They should not be cited or distributed as the published version of this manuscript.

BMJ Open is an open access journal and the full, final, typeset and author-corrected version of record of the manuscript is available on our site with no access controls, subscription charges or pay-per-view fees (http://bmjopen.bmj.com).

If you have any questions on BMJ Open's open peer review process please email info.bmjopen@bmj.com

BMJ Open

Magnetic Resonance Imaging to Assess the Neurodevelopmental Impact of Environment in the Early Years (MINE): A Prospective Cohort Study Protocol from a Low, Middle-Income Country

Journal:	*BMJ Open*
Manuscript ID	bmjopen-2022-070283
Article Type:	Protocol
Date Submitted by the Author:	17-Nov-2022
Complete List of Authors:	Surani, Zoya; The Aga Khan University, Department of Paediatrics and Child Health<br>Parkar, Sadia; The Aga Khan University, Department of Paediatrics and Child Health<br>Jafri, Sidra Kaleem; The Aga Khan University, Department of Paediatrics and Child Health<br>Afshan, Gul; The Aga Khan University, Department of Paediatrics and Child Health<br>Elahi, Kinza; The Aga Khan University, Department of Paediatrics and Child Health<br>Hussain, Syed Uzair; The Aga Khan University, Department of Paediatrics and Child Health<br>Hilal, Kiran; The Aga Khan University, Department of Radiology<br>Hoodbhoy, Zahra; The Aga Khan University, Department of Paediatrics and Child Health
Keywords:	PAEDIATRICS, Paediatric neurology < PAEDIATRICS, Community child health < PAEDIATRICS, Magnetic resonance imaging < RADIOLOGY & IMAGING, PUBLIC HEALTH

SCHOLARONE™
Manuscripts

TITLE: Magnetic Resonance **I**maging to Assess the **N**eurodevelopmental Impact of **E**nvironment in the Early Years (MINE): A Prospective Cohort Study Protocol from a Low, Middle-Income Country

Authors: Zoya Surani[1], Sadia Parkar[1], Sidra Kaleem Jafri[1], Gul Afshan[1], Kinza Elahi[1], Syed Uzair Hussain[1], Kiran Hilal[2], Zahra Hoodbhoy[1]

[1]Department of Paediatrics and Child Health, The Aga Khan University, Karachi, Pakistan
[2]Department of Radiology, The Aga Khan University, Karachi, Pakistan

Corresponding author: Dr. Sidra Kaleem Jafri
Address: Stadium Road, P.O. Box 3500, Karachi 74800, Pakistan
Email address: sidra.kaleem@aku.edu

Author email addresses:
Zoya Surani (zoyasurani@gmail.com), Sadia Parkar (sadia.parkar@aku.edu), Sidra Kaleem Jafri (sidra.kaleem@aku.edu), Gul Afshan (afshan.gul@aku.edu), Kinza Elahi (kinza.elahi@aku.edu), Syed Uzair Hussain (syeduzair.hussain@aku.edu), Kiran Hilal (kiran.hilal@aku.edu), Zahra Hoodbhoy (zahra.hoodbhoy@aku.edu)

Word count: 3,153

For peer review only

ABSTRACT

Introduction: Environmental and psychosocial adversities negatively impact children's developmental outcomes. When these factors are experienced in early childhood–a sensitive period of development–the developing brain can be altered. While these associations have been drawn in high-income countries, it is necessary to understand child growth, neurodevelopment, and the role of environmental factors in developmental trajectories in low-income settings. The objective of this study is to longitudinally assess how demographic factors, maternal health, maternal development, and child health are related to child development on a behavioral, cognitive, and neuroimaging level in low-socioeconomic communities.

Methods and analysis: Mother-child dyads will be identified in the peri-urban field sites of Rehri Goth and Ibrahim Hyderi, Karachi, Pakistan. Dyads will undergo yearly assessments for four years beginning when the child is 1 month, 3 months, or 6 months of age (depending on group assignment). Maternal assessments include anthropometry, behavioral, cognitive, and developmental assessments (Edinburgh Postnatal Depression Scale; Parenting Stress Index; Maternal Autonomy Index; Hurt, Insult, Threaten, Scream Tool; Reynolds Intellectual Assessment Scales), and biological samples collection (breastmilk, blood, stool, hair). Children's assessments include anthropometry, developmental assessments (Global Scales for Early Development; RIAS), MRI brain assessments and biological samples collection (blood, stool, hair). Using cross-sectional and longitudinal data with statistical analysis tools, associations will be quantified between brain structure (magnetic resonance imaging) and connectivity (resting state connectivity and diffusion tensor imaging), general cognitive skills (RIAS; GSED), and environmental influences (nutrition via biological samples, maternal mental health via questionnaires) through repeated measures ANOVA tests and chi-square tests. Quantile regression and cortical analyses will be conducted to understand how demographic factors are related to the associations found.

Ethics and dissemination: The study has received ethical approval from the Aga Khan University Ethics Review Committee. The study's findings will be disseminated through scientific publications and project summaries for the participants.

Strength and limitations of this study:

- This is one of the first studies to longitudinally assess children from early infancy (1 month) to 4 years of age with several behavioral, cognitive, biological, and neuroimaging measures in low resource settings.
- There will not be a direct control group in this study.
- A sample size calculation was not completed to determine the power of the study.

Keywords: paediatrics, paediatric neurology, community child health, magnetic resonance imaging, public health

For peer review only

INTRODUCTION

Worldwide, an estimated 250 million children under the age of 5 years fail to meet their developmental potential due to the exposure to early life adversities, such as malnutrition, psychosocial and environmental stress, and neglect (1). Many of these children live in low- and middle-income countries (LMICs), where there is often a profound confluence of negative environmental and psychosocial factors (2). The first 1000 days of a child's life are widely recognized as a sensitive period of development, where the structural and functional architecture of the brain is laid, and lifelong patterns of physical, cognitive, and socio-emotional health are established (3,4).

Throughout this period, the fetal and infant's brain increases almost three-fold in volume, and its extensive neuroarchitecture matures through processes including neurogenesis, migration, synaptogenesis, and myelination (5). This transformation is fueled by the mother and child's nutritional and energy resources, and shaped by cascades of genetic and environmental interactions–modulated through psychosocial and caregiving relationships (6,7). Previous neuroimaging studies provide clear evidence of how these factors can alter the developing brain, particularly when these adversities are experienced during the early life years.

Infants are inherently competent in their ability to initiate relationships, explore, seek meaning, and learn, but are also vulnerable and depend entirely on caregivers for their survival, emotional security, modeling of behaviors, and to teach them the norms of the physical and socio-cultural world that they inhabit (8,9). This sensitive period, then, is a double-edged sword. While positive and enriching environments can promote healthy brain development (10–12), neglect and abuse can impair maturing brain systems and disrupt cognitive and behavioral outcomes (13–15). Early adversities disrupt normative developmental trajectories with lifelong consequences, including impaired intellectual functioning, depression, and other mental health disorders (15–17). Therefore, adversity and resource inequality not only contribute to poor brain development but are also an outcome of it, potentially driving a vicious intergenerational cycle (18).

While existing literature on this topic has largely focused on understanding the neurodevelopmental impact of environmental factors as they exist in high-income areas, literature is evolving to include more data from LMICs–as understanding the nature of adversities in different environments is an important consideration, particularly in LMICs where the burden to do so is greater. Of the studies that aim to understand neurodevelopment, there has been a recent focus on using magnetic resonance imaging (MRI) as the preferred modality of neuroimaging due to its anatomical tissue contract, ability to visualize anatomical structure and physiological function, and its safety in use (19). By using MRI to image young children, there is a greater ability to visualize and understand brain plasticity, and development of perceptual and cognitive skills (19).

The two major studies conducted on this topic in LMICs were completed in Bangladesh and India. In the Bangladesh Early Adversity Neuroimaging study, associations between psychosocial risks (maternal distress, caregiving experiences), biological risks (growth, inflammation), developmental outcomes (Mullen Scales, Wechsler Preschool and Primary Scale, Bayley Scales), and neurodevelopment (electroencephalography, MRI) were assessed in children aged 6 months to 7 years (20). In this study, as well as another conducted in India to understand

For peer review only

neurodevelopmental outcomes in infants up to 18 months of age, researchers found that adverse factors present in LMIC environments are associated with outcomes on the cognitive and neural level (20,21). These studies highlight the importance of using MRI in LMICs to investigate the impact of these environments.

Despite the presence of studies in LMICs aimed at explaining children's neurodevelopment in these environments, there is a decreased understanding of the mechanisms involved in how and why children's outcomes are impacted. These are important considerations because before effective interventions can be designed to minimize adversities and their impact on children's development and neurodevelopment, it is necessary to understand the role of environmental factors. Knowing that the early years are a time of immense brain development and extremely susceptible to factors in the environment, it is necessary to comprehensively map children's growth and development–assessing both environmental factors and brain structure continuously.

The primary objective of the current study is to longitudinally map trajectories of child growth and neurodevelopment from early infancy (1 month) to 4 years of age using longitudinal MRI scanning and neurodevelopmental assessments in Karachi, Pakistan. The secondary objective of this study is to investigate the relationships between modifiable environmental factors (e.g., early child nutrition, maternal stress) with evolving brain structure, brain function, and maturing cognitive skills. We hypothesize that children with exposure to conditions like maternal and child malnutrition and adverse childhood experiences will have poor development with macro-level structural brain differences in comparison to children who were not exposed to these conditions.

METHODS AND ANALYSIS

Setting

The study will be conducted at Rehri Goth (RG) and Ibrahim Hyderi (IH), which are peri-urban communities in Karachi, Pakistan. Maintained by the Department of Paediatrics and Child Health at The Aga Khan University (AKU), these sites have quarterly household surveillance for key maternal and child health indicators along with antenatal care, immunisation and physician services for children under the age of 5 years available at the primary health care clinic (PHC) within the community.

Design

This study is a longitudinal cohort study and will be built upon the Pregnancy Risk Stratification Innovation and Measurement Alliance (PRiSMA) study (ERC Number: 2021-5920-15518). The PRiSMA study is a longitudinal study that enrolls pregnant women within the first 20 weeks of gestation and follows them and their newborns until one year after delivery. The current study will map trajectories of child growth and neurodevelopment from 1 month to 4 years of age. Additionally, the reporting of this study conforms to the STROBE statement (22).

Duration

The study will be conducted over a 3.5-year period (May 2022 through December 2026).

Inclusion and exclusion criteria

The inclusion criteria are:

- All singleton children 1 month, 3 months, or 6 months of age

For peer review only

The exclusion criteria are:

- Infants with congenital anomalies (established on clinical visits)
- Infants with history of hypoxic-ischemic insult (delayed cry, requiring oxygen and hospitalization)
- Maternal usage of anti-depressive or anti-epileptic medications during pregnancy
- Fetal exposure to maternal smoking (cigarettes)
- Ultrasound abnormalities (e.g., ventriculomegaly, renal agenesis, gastrointestinal structural anomalies, etc.)
- Conditions like preeclampsia, high blood pressure, or diabetes mellitus in pregnancy

Sample size calculation

A formal sample size calculation to obtain the power of the study was not calculated. We aimed to collect data on 150 mother-child dyad pairs over the study duration. However, keeping attrition of 40% due to identified challenges (e.g., difficulty to obtain follow-up data due to family relocation, family refusal, etc.), approximately 250 mother-child dyads will be recruited.

Recruitment procedure

Post-delivery, women enrolled in the PRiSMA project who meet the inclusion criteria will be approached at their home by research staff and explained the purpose of the current study. The mother-child dyad will be chosen through simple random sampling to reduce any sampling bias. Once written informed consent is provided by the parent/guardian, the enrollment visit will be performed, and a unique study participant identification number (ID) will be assigned to the infant and mother, where they will both have the same ID number, but labeled with "infant" or "mother" to distinguish them (e.g., infant ID: Infant-1234, mother ID: Mother-1234). The participants will be recruited in three groups: Group 1 include 50 mother-infant dyads with infants that are 1 month +/- 1 week of age, Group 2 will include 100 mother-infant dyads with infants that are 3 months +/- 1 week of age, and Group 3 will include 100 mother-infant dyads with infants that are 6 months +/- 1 week of age. This split will help the team obtain images and samples at different timepoints during the early childhood period without the need for repetitive sampling of any one mother-child dyad.

The study involves the mother-child dyads completing four visits over the course of 3.5 years. Each visit will comprise of the child's MRI brain scan, followed by the collection of information and biological samples. For the MRI scan, the family will be transported to AKU, accompanied by members of the research team. Once the scan is completed, the demographic information, maternal physical and mental health assessments, the child's history, child physical health and developmental assessments, and biological samples will be collected on the following day at the primary health center (PHC) at the study site. This flow will be adapted to ensure effective use of resources once the MRI scan is successfully performed. Additionally, quality control programs are in place for data collection to ensure there are no changes in the measurement of variables over the study's duration.

During the study period, each child will have at least three MRI scans at different ages of growth to assess brain development. If any brain abnormalities are found, these children will be referred to the local health care facilities, however, the provision of these services is not part of the study.

For peer review only

MRI brain scan analysis will be completed at Brown University's MRI Research Facility, Rhode Island, USA. Blood and stool samples will be analyzed at the Infectious Disease Research Lab at AKU in Karachi, Pakistan, while breast milk will be analyzed at Karachi University, Karachi, Pakistan.

A summary of the visits is provided in Figure 1 and descriptions of each measure to be collected are provided in the next section.

(Figure 1)

Measures of outcomes

1) Demographics and history
    - *Demographic/socioeconomic information*
      Demographic and socioeconomic data will be collected at all visits (1-4). The data will include detailed maternal pregnancy history (gravidity, live births, pregnancy spacing, maternal age at each pregnancy), family composition (child ages and sex), socioeconomic and household asset information (mother and father education and occupation, religion, caste, household income, home size and number of rooms, toilet type, electricity access, number of mobile phones, housing type, cooking fuel, home, land and/or animal ownership, and transportation access.
    - *Child's history*
      A detailed assessment regarding the child's well-being, co-morbidities if any, major hospitalizations, child diarrhea events, antibiotic use, and vaccination history will be collected at visit 1. Labor and delivery information, as well as birth anthropometry, will be obtained from the PRiSMA records.

2) Maternal assessments
    - *Physical health assessments*
        i. <u>Anthropometric measurements</u>: Assessment of maternal anthropometry i.e. weight and height will be recorded using a calibrated weighing scale (Seca 874 Mobile Flat Scale) and measuring tape (Seca 212 Measuring Tape) respectively at visit 1.
        ii. <u>Biological sample collection</u>: Lab investigations of serum iron, total iron-binding capacity (TIBC), red blood cell (RBC) folate, Vitamin B-12 levels, Vitamins A and D, blood lead levels, and thyroid stimulating hormone (TSH) will be at visit 1. A sample of the mother's hair will be collected at visit 1 and 3 to assess for cortisol. A breast milk sample (20 mL) will be collected from the mother at visit 1 and the composition (general macronutrient (protein, fat, and carbohydrate content, as well as cholesterol, choline, and vitamin B content) will be determined. Stool sample for fecal microbiome analysis will be collected from the mother at visit 1.
    - *Behavioral and cognitive assessments*
      Behavioral and cognitive assessments will be administered at visit 1.
        i. <u>Maternal mental health</u> will be assessed by using Urdu translations of the Edinburgh Postnatal Depression Scale (EPDS) (23), Parenting Stress Index

For peer review only

(PSI) long-form (24), and through collecting the hours of maternal rest per day. The EPDS and PSI have been locally validated (25–27).

ii. Maternal autonomy, family violence, addiction, and neglect will be assessed using the Maternal Autonomy Index (28), and Urdu translations of the Hurt, Insult, Threaten, and Scream (HITS) Tool (29) for Intimate Partner Violence Screening for adults and has been locally validated (30,31).

- *Developmental assessment*

The Reynolds Intellectual Assessment Scales (RIAS) (32) will be used to assess the mother's development at visit 1. The RIAS measures general intelligence and nonverbal intelligence, and is administered to individuals ages 3 to 94. The RIAS has been locally validated (33).

3) Child assessments

- *Physical health assessments*

i. Anthropometric measurements: Weight, height, and head circumference of the child will be recorded using a calibrated weighing scale (Seca 334 Mobile Digital Baby Scale), infant measuring board (Seca 416 Infantometer), and measuring tape (Seca 212 Measuring Tape) respectively. Mid-upper arm circumference will also be measured for all children. Z-scores will be calculated using appropriate growth curves and standards for weight, length/height, weight-for-length/height, and head circumference-for-age. Child anthropometric measurements will be collected yearly (visits 1-4).

ii. Biological sample collection: Lab investigations of serum iron, TIBC, RBC folate, Vitamin B-12 levels, Vitamins A and D, blood lead levels, and TSH will be completed yearly (visits 1-4). Stool samples will be collected yearly (visits 1-4) and will be assessed for fecal microbiome (visits 1 and 3) and helminth (visits 2, 3, and 4). Hair sample to assess cortisol levels will be collected at visits 3 and 4.

- *Developmental assessments*

The Global Scales for Early Development (GSED) (34) (0 to 6 years) and Reynolds Intellectual Assessment Scales (RIAS) (non-verbal) (3 to 94 years) tools will be used to assess the child's development. The GSED will be administered at all visits (1-4), while the RIAS will only be administered to the child at visit 4 (when the child is >3 years old).

i. GSED: The World Health Organization (WHO) has developed and is currently validating the GSED, which can be used to assess children's development in the early years of life across the globe, including in Pakistan. As part of this scale, the short form (SF) and long-form (LF) will be used. Both SF and LF are unidimensional and provide a single age-adjusted score that represents a child's level of development: The SF uses a caregiver-reported questionnaire while the LF is a directly administered (observed) tool assessing the child's abilities and behaviors.

ii. RIAS: The RIAS measures general intelligence and nonverbal intelligence, and is administered to individuals ages 3 to 94. As the participants will be >3 years old only at visit 4, the RIAS will be administered only at that visit.

- *MRI brain scanning*

For peer review only

The scan will be completed using a 3T MRI (Toshiba) machine. T1 and T2-weighted anatomical imaging, as well as quantitative (q)T1, qT2, myelin water fraction (MWF), multiple b-value diffusion, and resting-state connectivity (rsMRI), McDESPOT, Multishell DTI, and T1W MPRAGE will be collected.

For MRI brain scanning, the mother and child will be brought to AKU accompanied with members of the research team. Once the child appears to be in deep sleep (15-20 mins after the child went to sleep), the MRI will be performed. The child will then be secured in an immobilizer and transferred from their crib or bed to an MRI-compatible cart. The immobilizer will be used to reduce subtle body movement during the scan (e.g., movement from deep sleep breathing). The child's head will be carefully positioned, and a removable sound-insulating foam insert (Ultra Barrier HD Composite; American Micro Industries, Chambersburg, PA, USA) will be securely placed on the child's ears. The process will be facilitated by a research coordinator, and parents will be invited to stay with the infant to allay any anxiety. A pediatric pulse oximeter will be attached to the child's finger or toe to monitor the child during the scan. A research assistant will remain inside the scanning suite in case the child wakes up during the scan.
The scan will ideally be performed without sedation at any stage of the scan, using strategies developed by Dean et al (19). In case the child does not sleep, we will use minimally conscious sedation routinely employed in the pediatric population. For infants up to 3 months this includes chloral hydrate, 25mg per kg given orally, while for children 6 months and above, procedural sedation will be used which will be carried out by a credentialed physician and sedation nurse. Children requiring sedation will be admitted for pre, intra, and post sedation monitoring.

Patient and public involvement
Patients and the public were not involved in the design of this study. However, study participants will be informed regarding the study findings after the study results are published and their help will be enlisted to help with the dissemination of research findings.

Statistical analysis plan
Analysis of participant characteristics will be conducted using STATA version 17. Participant characteristics will be described using means and standard deviations for continuous variables (anthropometric measurements, maternal behavioral, cognitive, and developmental assessments scoring, and child developmental assessments scoring) and percentages for categorical variables (biological samples, demographics, and child's history information).

MRI brain analysis will be performed at Brown University MRI Research Facility (MRF), Rhode Island, U.S.A. The MRI and DTI brain files will be shared from AKUH with the MRF team via the Flywheel platform after the Data Transfer Agreement is signed.

Patterns of structural and functional development will be characterized, and associations will be conducted between cognitive outcomes and deviations associated with birth outcomes and environmental factors at various time points. Imaging data will be paired with neurocognitive assessments (GSED, RIAS, etc.) performed at predetermined intervals (GSED at visits 1-3, RIAS

For peer review only

at visit 4). We will quantify the associations (e.g., with a chi square test) between maternal and child health (via levels of micronutrients in the blood and breastmilk) on measures of fetal and infant growth and neurodevelopment. We will use repeated measures ANOVA tests to determine statistically significant differences between measures collected at various time points–child anthropometric measurements, biological samples, child developmental assessment scores, and child MRI scanning via resting-state connectivity patterns through fMRI and functional connectivity through DTI. Quantile regression analyses will be performed to understand if demographic factors (e.g., socioeconomic status) and/or child sex are related to any of the associations found between cognitive skills, environmental influences, and emerging neurobehavior. Cortical thickness analyses will be conducted to specifically understand the correlation between demographic factors and structural differences (via cortical thickness as found through T1W MRI).

As this is a longitudinal study, due to identified challenges that may result in a loss of follow-up, we have kept attrition at 40%, thus increasing our participant recruitment to 250 mother-child dyads for our ultimate sample size of 150 mother-child dyads. Additionally, during the analysis, if there is missing data, the data will be addressed appropriately to ensure the remaining objective and subjective data are able to be maximally used.

ETHICS AND DISSEMINATION

Ethics approval of the study was obtained from the Ethics Review Committee of Aga Khan University (Reference Number: 2022-7181-21831). Written informed consent will be voluntarily obtained from all participants, and participants will be informed of their complete right of refusal to initiate or withdraw at any stage of the study with no obligations. Consent for the biological samples will be obtained as part of the consent for participation in the study. Hospital data transfer policy will be employed, and all data collected and/or shared from participants will be de-identified.

The study's findings will be disseminated through scientific publications and medical education seminars. Participants will be informed of the study's findings through project summaries with the key findings included.

Acknowledgments: We thank our Aga Khan University Senior Research Assistants Sahiba Inayat, Adeeba Samreen, Shumaila Samito, our data collectors, and field team for their help in project management and development.

Author Contributions: ZS, SP, SK, GA, KE, SUH, KH, and ZH were involved in the concept, grant writing protocol development and intellectual inputs for this project. SK, ZH and KH provided expert advice and technical training. All authors contributed towards development of this manuscript.

Funding: This work was supported by The Bill & Melinda Gates Foundation (grant number: INV-004939).

Competing interests: None declared.

License Agreement: I, the Submitting Author Zoya Surani, has the right to grant and does grant on behalf of all authors of the Work (as defined in the below author licence), an exclusive licence and/or a non-exclusive licence for contributions from authors who are: i) UK Crown employees;

For peer review only

ii) where BMJ has agreed a CC-BY licence shall apply, and/or iii) in accordance with the terms applicable for US Federal Government officers or employees acting as part of their official duties; on a worldwide, perpetual, irrevocable, royalty-free basis to BMJ Publishing Group Ltd ("BMJ") its licensees and where the relevant Journal is co-owned by BMJ to the co-owners of the Journal, to publish the Work in BMJ Open and any other BMJ products and to exploit all rights, as set out in our licence.

The Submitting Author accepts and understands that any supply made under these terms is made by BMJ to the Submitting Author unless you are acting as an employee on behalf of your employer or a postgraduate student of an affiliated institution which is paying any applicable article publishing charge ("APC") for Open Access articles. Where the Submitting Author wishes to make the Work available on an Open Access basis (and intends to pay the relevant APC), the terms of reuse of such Open Access shall be governed by a Creative Commons licence – details of these licences and which Creative Commons licence will apply to this Work are set out in our licence referred to above.

For peer review only

REFERENCES

1. Prendergast AJ, Humphrey JH. The stunting syndrome in developing countries. Paediatr Int Child Health. 2014 Nov;34(4):250–65.

2. Johnson SB, Riis JL, Noble KG. State of the Art Review: Poverty and the Developing Brain. Pediatrics. 2016 Apr;137(4):e20153075.

3. Johnson MH. Functional brain development in humans. Nat Rev Neurosci. 2001 Jul;2(7):475–83.

4. Clouchoux C, Guizard N, Evans AC, du Plessis AJ, Limperopoulos C. Normative fetal brain growth by quantitative in vivo magnetic resonance imaging. Am J Obstet Gynecol. 2012 Feb;206(2):173.e1-8.

5. Houston SM, Herting MM, Sowell ER. The Neurobiology of Childhood Structural Brain Development: Conception Through Adulthood. Curr Top Behav Neurosci. 2014;16:3–17.

6. Stiles J, Jernigan TL. The basics of brain development. Neuropsychol Rev. 2010 Dec;20(4):327–48.

7. Rice D, Barone S. Critical periods of vulnerability for the developing nervous system: evidence from humans and animal models. Environ Health Perspect. 2000 Jun;108 Suppl 3:511–33.

8. Lally JR. For Our Babies. Teachers College Press; 2015.

9. Hofman A, Jaddoe VWV, Mackenbach JP, Moll HA, Snijders RFM, Steegers EAP, et al. Growth, development and health from early fetal life until young adulthood: the Generation R Study. Paediatr Perinat Epidemiol. 2004 Jan;18(1):61–72.

10. Dobbing J. THE INFLUENCE OF EARLY NUTRITION ON THE DEVELOPMENT AND MYELINATION OF THE BRAIN. Proc R Soc Lond B Biol Sci. 1964 Feb 18;159:503–9.

11. Swain JE, Lorberbaum JP, Kose S, Strathearn L. Brain basis of early parent-infant interactions: psychology, physiology, and in vivo functional neuroimaging studies. J Child Psychol Psychiatry. 2007 Apr;48(3–4):262–87.

12. Else-Quest NM, Hyde JS, Clark R. Breastfeeding, Bonding, and the Mother-Infant Relationship. Merrill-Palmer Q. 2003;49(4):495–517.

13. Rees S, Inder T. Fetal and neonatal origins of altered brain development. Early Hum Dev. 2005 Sep;81(9):753–61.

14. Feinberg I. Schizophrenia: caused by a fault in programmed synaptic elimination during adolescence? J Psychiatr Res. 1982 1983;17(4):319–34.

For peer review only

15. Farah MJ, Shera DM, Savage JH, Betancourt L, Giannetta JM, Brodsky NL, et al. Childhood poverty: specific associations with neurocognitive development. Brain Res. 2006 Sep 19;1110(1):166–74.

16. Bick J, Nelson CA. Early Adverse Experiences and the Developing Brain. Neuropsychopharmacol Off Publ Am Coll Neuropsychopharmacol. 2016 Jan;41(1):177–96.

17. Nelson CA, Gabard-Durnam LJ. Early Adversity and Critical Periods: Neurodevelopmental Consequences of Violating the Expectable Environment. Trends Neurosci. 2020 Mar;43(3):133–43.

18. Walker SP, Wachs TD, Grantham-McGregor S, Black MM, Nelson CA, Huffman SL, et al. Inequality in early childhood: risk and protective factors for early child development. Lancet Lond Engl. 2011 Oct 1;378(9799):1325–38.

19. Dean DC, Dirks H, O'Muircheartaigh J, Walker L, Jerskey BA, Lehman K, et al. Pediatric neuroimaging using magnetic resonance imaging during non-sedated sleep. Pediatr Radiol. 2014 Jan;44(1):64–72.

20. Jensen SKG, Kumar S, Xie W, Tofail F, Haque R, Petri WA, et al. Neural correlates of early adversity among Bangladeshi infants. Sci Rep. 2019 Mar 5;9(1):3507.

21. Aker K, Thomas N, Adde L, Koshy B, Martinez-Biarge M, Nakken I, et al. Prediction of outcome from MRI and general movements assessment after hypoxic-ischaemic encephalopathy in low-income and middle-income countries: data from a randomised controlled trial. Arch Dis Child - Fetal Neonatal Ed. 2022 Jan;107(1):32–8.

22. von Elm E, Altman DG, Egger M, Pocock SJ, Gøtzsche PC, Vandenbroucke JP. The Strengthening the Reporting of Observational Studies in Epidemiology (STROBE) Statement: Guidelines for Reporting Observational Studies. Ann Intern Med. 2007 Oct 16;147(8):573–7.

23. Cox JL, Holden JM, Sagovsky R. Detection of postnatal depression. Development of the 10-item Edinburgh Postnatal Depression Scale. Br J Psychiatry J Ment Sci. 1987 Jun;150:782–6.

24. Loyd BH, Abidin RR. Revision of the Parenting Stress Index. J Pediatr Psychol. 1985 Jun 1;10(2):169–77.

25. Humayun A, Haider II, Imran N, Iqbal H, Humayun N. Antenatal depression and its predictors in Lahore, Pakistan. East Mediterr Health J. 2013 Apr 1;19(04):327–32.

26. Atif M, Halaki M, Chow CM, Raynes-Greenow C. Risk factors of paternal postnatal depression in Pakistan: Findings from an urban sample. Nurs Health Sci. 2022;24(3):618–24.

27. Ahmad S, Khanam SJ. Parenting Stress Related to Behavior Problems of Children with Hearing Impairment. J Pak Psychiatr Soc. 2015 Jan;12(1):51–2.

For peer review only

28. IndiKit. Women's Autonomy Index [Internet]. 2021 [cited 2022 Oct 20]. Available from: https://www.indikit.net/indicator/327-women-s-autonomy-index

29. Sherin KM, Sinacore JM, Li XQ, Zitter RE, Shakil A. Hits: A short domestic violence screening tool for use in a family practice setting. Fam Med. 1998;30(7):508–12.

30. Nafees R, Safdar Z, Baig FS, Sher SJ. Knowledge and Perception Among Gynecologists Regarding Screening of Domestic Violence against Women. BioMedica. 2021 Dec 30;37(4):253–8.

31. Ahmad A, Aziz M, Anjum G, Mir FV. Intimate Partner Violence and Psychological Distress: Mediating Role of Stockholm Syndrome. :17.

32. C.R. Reynolds, Kamphaus, R.W. Reynolds Intellectual Assessment Scales, Second Edition (RIAS-2). Psychol Assess Resour Inc. 2015;

33. Riaz Z, Shahzad S, Ahmad R, Khanam SJ. Psychological Adjustment among Intellectually Gifted Secondary School Children. Pak J Psychol [Internet]. 2013 Dec 31 [cited 2022 Oct 31];44(2). Available from: http://www.pjpku.com/index.php/pjp/article/view/100

34. GSED Team. The Global Scale for Early Development (GSED). Early Child Matters. 2019;14:80–4.

For peer review only

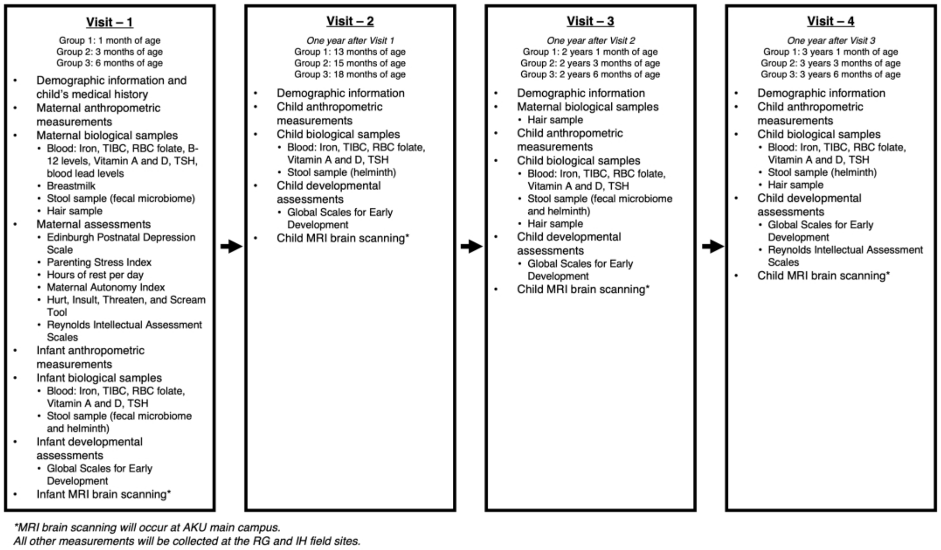

Figure 1: Summary of measures collected in each of the four visits from both the mother and child.

338x190mm (300 x 300 DPI)

Reporting checklist for cohort study.

Based on the STROBE cohort guidelines.

Instructions to authors

Complete this checklist by entering the page numbers from your manuscript where readers will find each of the items listed below.

Your article may not currently address all the items on the checklist. Please modify your text to include the missing information. If you are certain that an item does not apply, please write "n/a" and provide a short explanation.

Upload your completed checklist as an extra file when you submit to a journal.

In your methods section, say that you used the STROBE cohortreporting guidelines, and cite them as:

von Elm E, Altman DG, Egger M, Pocock SJ, Gotzsche PC, Vandenbroucke JP. The Strengthening the Reporting of Observational Studies in Epidemiology (STROBE) Statement: guidelines for reporting observational studies.

		Reporting Item	Page Number
**Title and abstract**			
Title	#1a	Indicate the study's design with a commonly used term in the title or the abstract	1
Abstract	#1b	Provide in the abstract an informative and balanced summary of what was done and what was found	2
**Introduction**			
Background / rationale	#2	Explain the scientific background and rationale for the investigation being reported	3
Objectives	#3	State specific objectives, including any prespecified hypotheses	4
**Methods**			
Study design	#4	Present key elements of study design early in the paper	4

For peer review only

Setting	#5	Describe the setting, locations, and relevant dates, including periods of recruitment, exposure, follow-up, and data collection	4
Eligibility criteria	#6a	Give the eligibility criteria, and the sources and methods of selection of participants. Describe methods of follow-up.	4-5
Eligibility criteria	#6b	For matched studies, give matching criteria and number of exposed and unexposed	n/a, not completing a match study
Variables	#7	Clearly define all outcomes, exposures, predictors, potential confounders, and effect modifiers. Give diagnostic criteria, if applicable	6-8
Data sources / measurement	#8	For each variable of interest give sources of data and details of methods of assessment (measurement). Describe comparability of assessment methods if there is more than one group. Give information separately for for exposed and unexposed groups if applicable.	6-8
Bias	#9	Describe any efforts to address potential sources of bias	5
Study size	#10	Explain how the study size was arrived at	5
Quantitative variables	#11	Explain how quantitative variables were handled in the analyses. If applicable, describe which groupings were chosen, and why	6-8
Statistical methods	#12a	Describe all statistical methods, including those used to control for confounding	
8-9			
Statistical methods	#12b	Describe any methods used to examine subgroups and interactions	8-9
Statistical methods	#12c	Explain how missing data were addressed	9
Statistical methods	#12d	If applicable, explain how loss to follow-up was addressed	9
Statistical methods	#12e	Describe any sensitivity analyses	

For peer review only

n/a

Results

Participants	#13a	Report numbers of individuals at each stage of study—eg numbers potentially eligible, examined for eligibility, confirmed eligible, included in the study, completing follow-up, and analysed. Give information separately for for exposed and unexposed groups if applicable.	n/a, this is a protocol manuscript (so results are not available yet)
Participants	#13b	Give reasons for non-participation at each stage	n/a
Participants	#13c	Consider use of a flow diagram	

n/a

Descriptive data	#14a	Give characteristics of study participants (eg demographic, clinical, social) and information on exposures and potential confounders. Give information separately for exposed and unexposed groups if applicable.	n/a
Descriptive data	#14b	Indicate number of participants with missing data for each variable of interest	

n/a

Descriptive data	#14c	Summarise follow-up time (eg, average and total amount)	

n/a

Outcome data	#15	Report numbers of outcome events or summary measures over time. Give information separately for exposed and unexposed groups if applicable.	

n/a

Main results	#16a	Give unadjusted estimates and, if applicable, confounder-adjusted estimates and their precision (eg, 95% confidence interval). Make clear which confounders were adjusted for and why they were included	n/a
Main results	#16b	Report category boundaries when continuous variables were categorized	n/a
Main results	#16c	If relevant, consider translating estimates of relative risk into absolute risk for a meaningful time period	

For peer review only

n/a			
Other analyses	#17	Report other analyses done—eg analyses of subgroups and interactions, and sensitivity analyses	n/a
**Discussion**			
Key results	#18	Summarise key results with reference to study objectives	n/a, this is a protocol manuscript
Limitations	#19	Discuss limitations of the study, taking into account sources of potential bias or imprecision. Discuss both direction and magnitude of any potential bias.	n/a
Interpretation	#20	Give a cautious overall interpretation considering objectives, limitations, multiplicity of analyses, results from similar studies, and other relevant evidence.	n/a
Generalisability	#21	Discuss the generalisability (external validity) of the study results	n/a
**Other Information**			
Funding	#22	Give the source of funding and the role of the funders for the present study and, if applicable, for the original study on which the present article is based	9

The STROBE checklist is distributed under the terms of the Creative Commons Attribution License CC-BY. This checklist was completed on 16. November 2022 using https://www.goodreports.org/, a tool made by the EQUATOR Network in collaboration with Penelope.ai

For peer review only

BMJ Open

Methods to Image and Assess the Neurodevelopmental Impact of Environment in the Early Years (MINE): A Prospective Cohort Study Protocol from a Low, Middle-Income Country

Journal:	*BMJ Open*
Manuscript ID	bmjopen-2022-070283.R1
Article Type:	Protocol
Date Submitted by the Author:	13-Apr-2023
Complete List of Authors:	Surani, Zoya; The Aga Khan University, Department of Paediatrics and Child Health<br>Parkar, Sadia; The Aga Khan University, Department of Paediatrics and Child Health<br>Afshan, Gul; The Aga Khan University, Department of Paediatrics and Child Health<br>Elahi, Kinza; The Aga Khan University, Department of Paediatrics and Child Health<br>Hoodbhoy, Zahra; The Aga Khan University, Department of Paediatrics and Child Health<br>Hilal, Kiran; The Aga Khan University, Department of Radiology<br>Jafri, Sidra Kaleem; The Aga Khan University, Department of Paediatrics and Child Health
**Primary Subject Heading**:	Paediatrics
Secondary Subject Heading:	Public health
Keywords:	PAEDIATRICS, Paediatric neurology < PAEDIATRICS, Community child health < PAEDIATRICS, Magnetic resonance imaging < RADIOLOGY & IMAGING, PUBLIC HEALTH

SCHOLARONE™
Manuscripts

TITLE: Methods to **I**mage and Assess the **N**eurodevelopmental Impact of **E**nvironment in the Early Years (MINE): A Prospective Cohort Study Protocol from a Low, Middle-Income Country

Authors: Zoya Surani[1], Sadia Parkar[1], Gul Afshan[1], Kinza Elahi[1], Zahra Hoodbhoy[1], Kiran Hilal[2], Sidra Kaleem Jafri[1]

[1]Department of Paediatrics and Child Health, The Aga Khan University, Karachi, Pakistan
[2]Department of Radiology, The Aga Khan University, Karachi, Pakistan

Corresponding author: Dr. Sidra Kaleem Jafri
Address: Stadium Road, P.O. Box 3500, Karachi 74800, Pakistan
Email address: sidra.kaleem@aku.edu

Author email addresses:
Zoya Surani (zoyasurani@gmail.com), Sadia Parkar (sadia.parkar@aku.edu), Gul Afshan (afshan.gul@aku.edu), Kinza Elahi (kinza.elahi@aku.edu), Zahra Hoodbhoy (zahra.hoodbhoy@aku.edu), Kiran Hilal (kiran.hilal@aku.edu), Sidra Kaleem Jafri (sidra.kaleem@aku.edu)

Word count: 3873

For peer review only

ABSTRACT

Introduction: Environmental and psychosocial adversities negatively impact children's developmental outcomes. When these factors are experienced in early childhood–a sensitive period of development–the developing brain can be altered. While these associations have been drawn in high-income countries, it is necessary to understand child growth, neurodevelopment, and the role of environmental factors in developmental trajectories in low-income settings. The objective of this study is to longitudinally assess how demographic factors, maternal health, maternal development, and child health are related to child development on a behavioral, cognitive, and neuroimaging level in low-socioeconomic communities.

Methods and analysis: Mother-child dyads will be identified in the peri-urban field sites of Rehri Goth and Ibrahim Hyderi, Karachi, Pakistan. Dyads will undergo yearly assessments for four years beginning when the child is 1 month, 3 months, or 6 months of age (depending on group assignment). Maternal assessments include anthropometry, behavioral, cognitive, and developmental assessments (Edinburgh Postnatal Depression Scale; Parenting Stress Index; Maternal Autonomy Index; Hurt, Insult, Threaten, Scream Tool; Reynolds Intellectual Assessment Scales), and biological samples collection (breastmilk, blood, stool, hair). Children's assessments include anthropometry, developmental assessments (Global Scales for Early Development; RIAS), MRI brain assessments, and biological sample collection (blood, stool, hair). Using cross-sectional and longitudinal data with statistical analysis tools, associations will be quantified between brain structure (magnetic resonance imaging) and connectivity (resting state connectivity and diffusion tensor imaging), general cognitive skills (RIAS, GSED), and environmental influences (nutrition via biological samples, maternal mental health via questionnaires) through repeated measures ANOVA tests and chi-square tests. Quantile regression and cortical analyses will be conducted to understand how demographic factors are related to the associations found.

Ethics and dissemination: The study has received ethical approval from the Aga Khan University Ethics Review Committee. The study's findings will be disseminated through scientific publications and project summaries for the participants.

Strength and limitations of this study:

- This is a longitudinal study that will assess various behavioral, cognitive, biological, and neuroimaging factors related to child development yearly for four years.
- There will not be a direct control group in this study.
- Though the sample size was calculated taking into account attrition that may occur over the study's timeline, a formal sample size calculation was not completed to determine the power of the study.

Keywords: paediatrics, paediatric neurology, community child health, magnetic resonance imaging, public health

For peer review only

INTRODUCTION

Worldwide, an estimated 250 million children under the age of 5 years fail to meet their developmental potential due to the exposure to early life adversities, such as malnutrition, psychosocial and environmental stress, and neglect (1). Many of these children live in low- and middle-income countries (LMICs), where there is often a profound confluence of negative environmental and psychosocial factors (2). The first 1000 days of a child's life are widely recognized as a sensitive period of development, where the structural and functional architecture of the brain is laid, and lifelong patterns of physical, cognitive, and socio-emotional health are established (3).

Throughout this period, the fetal and infant's brain increases almost three-fold in volume, and its extensive neuroarchitecture matures through processes including neurogenesis, migration, synaptogenesis, and myelination (4). This transformation is fueled by the mother and child's nutritional and energy resources, and shaped by cascades of genetic and environmental interactions–modulated through psychosocial and caregiving relationships (3,5). Previous neuroimaging studies provide clear evidence of how these factors can alter the developing brain, particularly when these adversities are experienced during the early life years (6).

Infants are inherently competent in their ability to initiate relationships, explore, seek meaning, and learn, but are also vulnerable and depend entirely on caregivers for their survival, emotional security, modeling of behaviors, and to teach them the norms of the physical and socio-cultural world that they inhabit (7). This sensitive period, then, is a double-edged sword. While positive and enriching environments can promote healthy brain development, neglect and abuse can impair maturing brain systems and disrupt cognitive and behavioral outcomes (8,9). Early adversities disrupt normative developmental trajectories with lifelong consequences, including impaired intellectual functioning, depression, and other mental health disorders (6,10). Therefore, adversity and resource inequality not only contribute to poor brain development but are also an outcome of it, potentially driving a vicious intergenerational cycle (11).

While existing literature on this topic has largely focused on understanding the neurodevelopmental impact of environmental factors as they exist in high-income areas, literature is evolving to include more data from LMICs–as understanding the nature of adversities in different environments is an important consideration, particularly in LMICs where the burden to do so is greater. Of the studies that aim to understand neurodevelopment, there has been a recent focus on using magnetic resonance imaging (MRI) as the preferred modality of neuroimaging due to its anatomical tissue contract, ability to visualize anatomical structure and physiological function, and its safety in use (12). By using MRI to image young children, there is a greater ability to visualize and understand brain plasticity, and development of perceptual and cognitive skills (13).

The two major studies conducted on this topic in LMICs were completed in Bangladesh and India. In the Bangladesh Early Adversity Neuroimaging study, associations between psychosocial risks (maternal distress, caregiving experiences), biological risks (growth, inflammation), developmental outcomes (Mullen Scales, Wechsler Preschool and Primary Scale, Bayley Scales), and neurodevelopment (electroencephalography, MRI) were assessed in children aged 6 months to 7 years (14). In this study, as well as another conducted in India to understand

For peer review only

neurodevelopmental outcomes in infants up to 18 months of age, researchers found that adverse factors present in LMIC environments are associated with outcomes on the cognitive and neural level (14,15). These studies highlight the importance of using MRI in LMICs to investigate the impact of these environments.

Despite the presence of studies in LMICs aimed at explaining children's neurodevelopment in these environments, there is a decreased understanding of the mechanisms involved in how and why children's outcomes are impacted. These are important considerations because before effective interventions can be designed to minimize adversities and their impact on children's development and neurodevelopment, it is necessary to understand the role of environmental factors. Knowing that the early years are a time of immense brain development and extremely susceptible to factors in the environment, it is necessary to comprehensively map children's growth and development–assessing both environmental factors and brain structure continuously in low-resource environments.

Pakistan is among the countries with the highest rates of malnutrition and its progress in perinatal, neonatal, and child health has been much slower than in other South Asian countries (16,17). Karachi, the largest metropolitan city in Pakistan and 12th largest city in the world with a linguistically, ethnically, and religiously diverse population is an excellent surrogate to study the impact of environment on a child's development. The primary objective of the current study is to longitudinally map trajectories of child growth and neurodevelopment from early infancy (1 month) to 4 years of age using longitudinal MRI scanning and neurodevelopmental assessments in Karachi, Pakistan. The secondary objective of this study is to investigate the relationships between modifiable environmental factors (e.g., early child nutrition, maternal stress) with evolving brain structure, brain function, and maturing cognitive skills. We hypothesize that children with exposure to conditions like maternal and child malnutrition and adverse childhood experiences will have poor development with macro-level structural brain differences in comparison to children who were not exposed to these conditions.

METHODS AND ANALYSIS

Setting

In Karachi, to facilitate the large number of patients per day, The Aga Khan University Hospital has created a massive network with 4 secondary care hospitals, 30+ medical centers, 290+ clinical laboratories, and 20+ pharmacies in over 120 cities, and also provides home health care services across Pakistan. Aga Khan University Hospital in Karachi is connected with many primary health care clinics (PHCs) in the peri-urban coastal regions of the city and has a 3T MRI machine available onsite. The PHCs are maintained by the Department of Paediatrics and Child Health at AKU, and have quarterly household surveillance for key maternal and child health indicators along with antenatal care, immunisation and physician services for children under the age of 5 years. For these reasons, the provision of services at PHCs connected with the larger tertiary hospital make this area an ideal setting for this study. The study will be conducted at two of these PHCs, Rehri Goth (RG) and Ibrahim Hyderi (IH), which have been operating in the communities for over a decade.

Design

For peer review only

This study is a longitudinal cohort study and will be built upon the Pregnancy Risk Stratification Innovation and Measurement Alliance (PRiSMA) study (ERC Number: 2021-5920-15518). The PRiSMA study is a longitudinal study that enrolls pregnant women within the first 20 weeks of gestation and follows them and their newborns until one year after delivery. The current study will map trajectories of child growth and neurodevelopment from 1 month to 4 years of age. Additionally, the reporting of this study conforms to the STROBE statement (18).

Duration

The study will be conducted over a 3.5-year period (May 2022 through December 2026).

Inclusion and exclusion criteria

The inclusion criteria are:

- All singleton infants aged 1 month, 3 months, or 6 months of age and their mothers

The exclusion criteria are:

- Infants with congenital anomalies (established on clinical visits)
- Infants with history of hypoxic-ischemic insult (any of the three: delayed cry, requiring oxygen, or hospitalization)
- Maternal usage of anti-depressive or anti-epileptic medications during pregnancy
- Fetal exposure to maternal smoking (cigarettes)
- Ultrasound abnormalities (e.g., ventriculomegaly, renal agenesis, gastrointestinal structural anomalies, etc.)
- Conditions like preeclampsia, high blood pressure, or diabetes mellitus in pregnancy

Sample size calculation

A formal sample size calculation to obtain the power of the study was not calculated. We aimed to collect data on 150 mother-child dyad pairs over the study duration, basing our sample size on what was found feasible in previous neuroimaging infant studies (14,15). However, keeping attrition of 40% due to identified challenges (e.g., difficulty to obtain follow-up data due to family relocation, family refusal, etc.), approximately 250 mother-child dyads will be recruited.

Recruitment procedure

A list of newborns will be shared by the PRiSMA study team on a monthly basis. All infants will be screened for eligibility, and eligible mothers enrolled in the PRiSMA project will be approached at their home by research staff and explained the purpose of the current study. The mother-child dyads will be chosen and approached through simple random sampling to reduce any sampling bias.

Once written informed consent is provided by the parent/guardian, the enrollment visit will be performed, and a unique study participant identification number (ID) will be assigned to the infant and mother, where they will both have the same ID number, but labeled with “infant” or “mother” to distinguish them (e.g., infant ID: Infant-1234, mother ID: Mother-1234). The participants will be recruited in three groups: Group 1 will include 100 mother-infant dyads with infants that are 1 month +≤ 30 days of age, Group 2 will include 100 mother-infant dyads with infants that are 3 months +≤ 30 days of age, and Group 3 will include 50 mother-infant dyads with infants that are 6 months +≤ 30 days of age. As neuroimaging scans in natural sleep are more likely to be successful in infants of younger age and MRI scans with sedation may be more challenging to

For peer review only

obtain enrollments for, the group sizes of the younger groups (1-month and 3-month) are larger than that of the older infants (6-months). This split will also allow the team to obtain images and samples at different timepoints during the early childhood period without the need for repetitive sampling of any one mother-child dyad.

The study involves the mother-child dyads completing four visits over the course of 3.5 years. Each visit will comprise of the child's MRI brain scan, followed by the collection of information and biological samples. For the MRI scan, the family will be transported to AKU, accompanied by members of the research team. Once the scan is completed (estimated to take between 30 minutes to 1 hour, depending on participant cooperation), the demographic information, maternal physical and mental health assessments, the child's history, child physical health and developmental assessments, and biological samples will be collected on the following day at the primary health center (PHC) at the study site. The collection of the assessments and samples will take approximately two hours. The collection procedure of non-MRI data will be adapted to ensure effective use of resources once the MRI scan is successfully performed. Additionally, quality control programs are in place for data collection to ensure there are no changes in the measurement of variables over the study's duration. Weekly quality checks will be conducted under the supervision of master trainers for all behavioral, cognitive, and developmental assessment tools used in the study. Routine quality assurance of scans will be performed by MRI technicians immediately after the scan is obtained and again prior to image preprocessing. The quality of biological samples will be checked by the lab team and frequent quality management of all collected data will be done by the Data Management Unit.

A summary of the visits is provided in Figure 1 and descriptions of each measure to be collected are provided in the next section.

Measures of outcomes

1) Demographics and history
   - *Demographic/socioeconomic information*
     Demographic and socioeconomic data will be collected at all visits (1-4). The data will include detailed maternal pregnancy history (gravidity, live births, pregnancy spacing, maternal age at each pregnancy), family composition (child ages and sex), socioeconomic and household asset information (mother and father education and occupation, religion, caste, household income, home size and number of rooms, toilet type, electricity access, number of mobile phones, housing type, cooking fuel, home, land and/or animal ownership, and transportation access).
   - *Child's history*
     A detailed assessment regarding the child's well-being, co-morbidities if any, major hospitalizations, child diarrhea events, antibiotic use, and vaccination history will be collected at all visits. Labor and delivery information, as well as birth anthropometry, will be obtained from the PRiSMA records.

2) Maternal assessments
   - *Physical health assessments*
     i. <u>Anthropometric measurements</u>: Assessment of maternal anthropometry i.e. weight and height will be recorded using a calibrated weighing scale (Seca 874

Mobile Flat Scale) and measuring tape (Seca 212 Measuring Tape), respectively, at all visits.

ii. Biological sample collection: Lab investigations of serum iron, total iron-binding capacity (TIBC), red blood cell (RBC) folate, Vitamin B-12 levels, Vitamins A and D, blood lead levels, and thyroid stimulating hormone (TSH) will be done at visit 1. A sample of the mother's hair will be collected at visit 1 and 3 to assess for cortisol. A breastmilk sample (20 mL) will be collected from the mother at visit 1 and the composition (general macronutrient (protein, fat, and carbohydrate content), as well as cholesterol, choline, and vitamin B content) will be determined. Stool sample for fecal microbiome analysis will be collected from the mother at visit 1.

- *Behavioral and cognitive assessments*

Behavioral and cognitive assessments will be administered at all visits.

i. Maternal mental health will be assessed by using Urdu translations of the Edinburgh Postnatal Depression Scale (EPDS) (19), Parenting Stress Index (PSI) long-form (20), and through collecting the hours of maternal rest per day. The EPDS and PSI have been locally validated (21–23).

ii. Maternal autonomy, family violence, addiction, and neglect will be assessed using Urdu-translated versions of the Maternal Autonomy Index (24), and the Hurt, Insult, Threaten, and Scream (HITS) Tool (25), which have been locally validated (26,27).

- *Developmental assessment*

The Reynolds Intellectual Assessment Scales (RIAS) (28) will be used to assess the mother's development at all visits. The RIAS measures general intelligence and nonverbal intelligence, and is administered to individuals ages 3 to 94. The RIAS has been locally validated (29).

3) Child assessments

- *Physical health assessments*

i. Anthropometric measurements: Weight, height, and head circumference of the child will be recorded using a calibrated weighing scale (Seca 334 Mobile Digital Baby Scale), infant measuring board (Seca 416 Infantometer), and measuring tape (Seca 212 Measuring Tape), respectively. Mid-upper arm circumference will also be measured for children in each enrollment group at every visit. The WHO Z-scores will be used for anthropometric interpretation and standards for weight, length/height, weight-for-length/height, and head circumference-for-age will be calculated. Child anthropometric measurements will be collected yearly (visits 1-4) and analyzed within their enrollment group and visit number.

ii. Biological sample collection: Lab investigations of serum iron, TIBC, RBC folate, Vitamin B-12 levels, Vitamins A and D, blood lead levels, and TSH will be completed yearly (visits 1-4). Stool samples will be collected yearly (visits 1-4) and will be assessed for fecal microbiome (visits 1 and 3) and helminth (visits 2, 3, and 4). Hair sample to assess cortisol levels will be collected at visits 3 and 4.

- *Developmental assessments*

For peer review only

The Global Scales for Early Development (GSED) (30) (0 to 6 years) and Reynolds Intellectual Assessment Scales (RIAS) (non-verbal) (3 to 94 years) tools will be used to assess the child's development. The GSED will be administered at all visits (1-4), while the RIAS will only be administered to the child at visit 4 (when the child is >3 years old).

i. GSED: The World Health Organization (WHO) has developed and is currently validating the GSED, which can be used to assess children's development in the early years of life across the globe, including in Pakistan. As part of this scale, the short form (SF) and long-form (LF) will be used. Both SF and LF are unidimensional and provide a single age-adjusted score that represents a child's level of development: The SF uses a caregiver-reported questionnaire while the LF is a directly administered (observed) tool assessing the child's abilities and behaviors.

ii. RIAS: The RIAS measures general intelligence and nonverbal intelligence, and is administered to individuals ages 3 to 94. As the participants will be >3 years old only at visit 4, the RIAS will be administered only at that visit.

- *MRI brain scanning*

MRI brain scanning will be completed using a 3T MRI (Toshiba) machine at every visit (1-4). The total scan time of the MRI scan is 32:27 minutes, which includes T1 and T2-weighted anatomical imaging, diffusion tensor imaging, functional MRI, and FLAIR sequences. The scan card with sequence and sequence timing is included in Figure 2.

For MRI brain scanning, the mother and child will be brought to AKU accompanied with members of the research team. Once the child appears to be in deep sleep (15-20 mins after the child went to sleep), the MRI will be performed. The child will then be secured in an immobilizer (MedVac Immobilization Bag, Infant Splint; MRI Med, Petaluma, CA, USA) and transferred from their crib or bed to an MRI-compatible cart. The immobilizer will be used to reduce subtle body movement during the scan (e.g., movement from deep sleep breathing). The child's head will be carefully positioned, and a removable sound-insulating foam insert (Ultra Barrier HD Composite; American Micro Industries, Chambersburg, PA, USA) will be securely placed on the child's ears. The process will be facilitated by a research coordinator, and parents will be invited to stay with the infant to allay any anxiety. A pediatric pulse oximeter will be attached to the child's finger or toe to monitor the child during the scan. A research assistant will remain inside the scanning suite in case the child wakes up during the scan.

Though the total scan time is 32:27 minutes, repeating sequences depending on motion artifacts may result in the scan lasting longer. With children scanned in natural sleep, there is a possibility that they may wake up during the scan. If this happens, the child will be removed from the scanner and nursed and swaddled prior to continuing the scan. The scan will ideally be performed without sedation at any stage of the scan, using strategies developed by Dean et al (13). In case the child does not sleep, we will use minimally conscious sedation routinely employed in the pediatric population. For infants up to 3 months this includes chloral hydrate, 25mg per kg given orally, while for children 6 months and above, procedural sedation will be used which will be carried out by a credentialed physician and sedation nurse. The sedation medication will be

For peer review only

titrated according to the child's weight and children requiring sedation will be admitted for pre, intra, and post sedation monitoring.

During the follow-up visits, though the imaging data may be difficult to acquire in unsedated toddlers, the scans will be initially attempted in natural sleep. The data will be carefully reviewed to exclude low quality data and data that exceeds our motion threshold. Scans with sedation will be used as a last resort and will be performed rarely due to possible risks that might occur with annual evaluations with sedation.

While the length of the MRI exam will be affected by various factors, including patient motion and machine problems or troubleshooting during the scan (i.e. scanner calibration), all MRI data will undergo routine quality assurance. MRI data will be checked immediately after acquisition to ensure 1) no abnormalities were found, 2) all sequences were completed, and 3) there was limited motion. The MRI machine will also be continuously checked to ensure it is running properly, and if there are any machine or software malfunctions, scans will not be completed during that time.

Patient and public involvement

Patients and the public were not involved in the design of this study. However, study participants will be informed regarding the study findings after the study results are published and their help will be enlisted to help with the dissemination of research findings.

Statistical analysis plan

Analysis of participant characteristics will be conducted using STATA version 17. Participant characteristics will be described using means and standard deviations for continuous variables (anthropometric measurements, maternal behavioral, cognitive, and developmental assessments scoring, and child developmental assessments scoring) and percentages for categorical variables (biological samples, demographics, and child's history information).

MRI brain analysis will be performed locally, as experts from the funding agency will train the local Data Management Unit team at AKU. MRI data will be quality checked using Horos and Mango visualization tools. Analysis of neuroimaging data will be done using the Flywheel platform and the embedded FreeSurfer, FSL, and MRtrix3 analysis tools. Data will be segmented, skull-stripped, scaled to standard space, and registered to age-matched templates that will be created locally and matched with existing templates (e.g., NIH). For structural data, estimates of total brain tissue volume, thickness, and surface area will be acquired. Functional MRI data will be preprocessed and motion corrected to analyze differences in resting-state activity (as the scans will occur in natural or sedated sleep). Fractional anisotropy values will be obtained from the diffusion imaging to understand the shape of diffusion and will be visualized with tractography.

Ultimately, patterns of structural and functional development will be characterized, and associations will be conducted between cognitive outcomes and deviations associated with birth outcomes and environmental factors at various time points. Imaging data will be paired with neurocognitive assessments (GSED, RIAS, etc.) performed at predetermined intervals (GSED at visits 1-3, RIAS at visit 4). We will quantify the associations (e.g., with a chi-square test) between maternal and child health (via levels of micronutrients in the blood and breastmilk) on measures of fetal and infant growth and neurodevelopment. We will use repeated measures ANOVA tests to determine statistically significant differences between measures collected at various time

For peer review only

points–child anthropometric measurements, biological samples, child developmental assessment scores, and child MRI scanning via volume estimates through structural MRI, resting-state connectivity patterns through fMRI, and functional connectivity through DTI. Quantile regression analyses will be performed to understand if demographic factors (e.g., socioeconomic status) and/or child sex are related to any of the associations found between cognitive skills, environmental influences, and emerging neurobehavior. Cortical thickness analyses will be conducted to specifically understand the correlation between demographic factors and structural differences (via cortical thickness as found through T1W MRI).

As this is a longitudinal study, due to identified challenges that may result in a loss of follow-up, we have kept attrition at 40%, thus increasing our participant recruitment to 250 mother-child dyads for our ultimate sample size of 150 mother-child dyads. Additionally, during the analysis, if there is missing data, the data will be addressed appropriately to ensure the remaining data are able to be maximally used.

ETHICS AND DISSEMINATION

Ethics approval of the study was obtained from the Ethics Review Committee of Aga Khan University (Reference Number: 2022-7181-21831). Written informed consent will be voluntarily obtained from all participants, and participants will be informed of their complete right of refusal to initiate or withdraw at any stage of the study with no obligations. Consent for the biological samples will be obtained as part of the consent for participation in the study. Hospital data transfer policy will be employed, and all data collected and/or shared from participants will be de-identified.

The study's findings will be disseminated through scientific publications and medical education seminars. Participants will be informed of the study's findings through project summaries with the key findings included. As this study may provide insight to factors in the environment that will influence child development, the key points from the study will be shared with stakeholders and policymakers, who can use the study's findings to plan interventions for the physical and mental health of mothers and children at national, regional and international levels.

Acknowledgments: We thank our Aga Khan University Senior Research Assistants Sahiba Inayat, Adeeba Samreen, Shumaila Samito, our data collectors, and field team for their help in project management and development.
Author Contributions: ZS, SP, GA, KE, KH, ZH and SK were involved in the concept, grant writing protocol development and intellectual inputs for this project. SK, ZH and KH provided expert advice and technical training. All authors contributed towards development of this manuscript.
Funding: This work was supported by The Bill & Melinda Gates Foundation (grant number: INV-004939).
Competing interests: None declared.

License Agreement: I, the Submitting Author Zoya Surani, has the right to grant and does grant on behalf of all authors of the Work (as defined in the below author licence), an exclusive licence and/or a non-exclusive licence for contributions from authors who are: i) UK Crown employees; ii) where BMJ has agreed a CC-BY licence shall apply, and/or iii) in accordance with the terms

For peer review only

applicable for US Federal Government officers or employees acting as part of their official duties; on a worldwide, perpetual, irrevocable, royalty-free basis to BMJ Publishing Group Ltd ("BMJ") its licensees and where the relevant Journal is co-owned by BMJ to the co-owners of the Journal, to publish the Work in BMJ Open and any other BMJ products and to exploit all rights, as set out in our licence.

The Submitting Author accepts and understands that any supply made under these terms is made by BMJ to the Submitting Author unless you are acting as an employee on behalf of your employer or a postgraduate student of an affiliated institution which is paying any applicable article publishing charge ("APC") for Open Access articles. Where the Submitting Author wishes to make the Work available on an Open Access basis (and intends to pay the relevant APC), the terms of reuse of such Open Access shall be governed by a Creative Commons licence – details of these licences and which Creative Commons licence will apply to this Work are set out in our licence referred to above.

For peer review only

REFERENCES

1. Black MM, Walker SP, Fernald LCH, Andersen CT, DiGirolamo AM, Lu C, et al. Early childhood development coming of age: science through the life course. The Lancet. 2017 Jan 7;389(10064):77–90.

2. Johnson SB, Riis JL, Noble KG. State of the Art Review: Poverty and the Developing Brain. Pediatrics. 2016 Apr;137(4):e20153075.

3. Likhar A, Patil MS. Importance of Maternal Nutrition in the First 1,000 Days of Life and Its Effects on Child Development: A Narrative Review. Cureus. 14(10):e30083.

4. Houston SM, Herting MM, Sowell ER. The Neurobiology of Childhood Structural Brain Development: Conception Through Adulthood. Curr Top Behav Neurosci. 2014;16:3–17.

5. Stiles J, Jernigan TL. The basics of brain development. Neuropsychol Rev. 2010 Dec;20(4):327–48.

6. Nelson CA, Gabard-Durnam LJ. Early Adversity and Critical Periods: Neurodevelopmental Consequences of Violating the Expectable Environment. Trends Neurosci. 2020 Mar;43(3):133–43.

7. Köster M, Kayhan E, Langeloh M, Hoehl S. Making Sense of the World: Infant Learning From a Predictive Processing Perspective. Perspect Psychol Sci. 2020 May;15(3):562–71.

8. Ilyka D, Johnson MH, Lloyd-Fox S. Infant social interactions and brain development: A systematic review. Neurosci Biobehav Rev. 2021 Nov 1;130:448–69.

9. Miguel PM, Pereira LO, Silveira PP, Meaney MJ. Early environmental influences on the development of children’s brain structure and function. Dev Med Child Neurol. 2019;61(10):1127–33.

10. Bick J, Nelson CA. Early Adverse Experiences and the Developing Brain. Neuropsychopharmacol Off Publ Am Coll Neuropsychopharmacol. 2016 Jan;41(1):177–96.

11. Walker SP, Wachs TD, Grantham-McGregor S, Black MM, Nelson CA, Huffman SL, et al. Inequality in early childhood: risk and protective factors for early child development. Lancet Lond Engl. 2011 Oct 1;378(9799):1325–38.

12. Copeland A, Silver E, Korja R, Lehtola SJ, Merisaari H, Saukko E, et al. Infant and Child MRI: A Review of Scanning Procedures. Front Neurosci [Internet]. 2021 [cited 2023 Apr 7];15. Available from: https://www.frontiersin.org/articles/10.3389/fnins.2021.666020

13. Dean DC, Dirks H, O’Muircheartaigh J, Walker L, Jerskey BA, Lehman K, et al. Pediatric neuroimaging using magnetic resonance imaging during non-sedated sleep. Pediatr Radiol. 2014 Jan;44(1):64–72.

14. Jensen SKG, Kumar S, Xie W, Tofail F, Haque R, Petri WA, et al. Neural correlates of early adversity among Bangladeshi infants. Sci Rep. 2019 Mar 5;9(1):3507.

For peer review only

15. Aker K, Thomas N, Adde L, Koshy B, Martinez-Biarge M, Nakken I, et al. Prediction of outcome from MRI and general movements assessment after hypoxic-ischaemic encephalopathy in low-income and middle-income countries: data from a randomised controlled trial. Arch Dis Child - Fetal Neonatal Ed. 2022 Jan;107(1):32–8.

16. Bhutta ZA, Hafeez A, Rizvi A, Ali N, Khan A, Ahmad F, et al. Reproductive, maternal, newborn, and child health in Pakistan: challenges and opportunities. Lancet Lond Engl. 2013 Jun 22;381(9884):2207–18.

17. Khan GN, Turab A, Khan MI, Rizvi A, Shaheen F, Ullah A, et al. Prevalence and associated factors of malnutrition among children under-five years in Sindh, Pakistan: a cross-sectional study. BMC Nutr. 2016 Nov 24;2(1):69.

18. von Elm E, Altman DG, Egger M, Pocock SJ, Gøtzsche PC, Vandenbroucke JP. The Strengthening the Reporting of Observational Studies in Epidemiology (STROBE) Statement: Guidelines for Reporting Observational Studies. Ann Intern Med. 2007 Oct 16;147(8):573–7.

19. Cox JL, Holden JM, Sagovsky R. Detection of postnatal depression. Development of the 10-item Edinburgh Postnatal Depression Scale. Br J Psychiatry J Ment Sci. 1987 Jun;150:782–6.

20. Loyd BH, Abidin RR. Revision of the Parenting Stress Index. J Pediatr Psychol. 1985 Jun 1;10(2):169–77.

21. Humayun A, Haider II, Imran N, Iqbal H, Humayun N. Antenatal depression and its predictors in Lahore, Pakistan. East Mediterr Health J. 2013 Apr 1;19(04):327–32.

22. Atif M, Halaki M, Chow CM, Raynes-Greenow C. Risk factors of paternal postnatal depression in Pakistan: Findings from an urban sample. Nurs Health Sci. 2022;24(3):618–24.

23. Ahmad S, Khanam SJ. Parenting Stress Related to Behavior Problems of Children with Hearing Impairment. J Pak Psychiatr Soc. 2015 Jan;12(1):51–2.

24. IndiKit. Women's Autonomy Index [Internet]. 2021 [cited 2022 Oct 20]. Available from: https://www.indikit.net/indicator/327-women-s-autonomy-index

25. Sherin KM, Sinacore JM, Li XQ, Zitter RE, Shakil A. Hits: A short domestic violence screening tool for use in a family practice setting. Fam Med. 1998;30(7):508–12.

26. Nafees R, Safdar Z, Baig FS, Sher SJ. Knowledge and Perception Among Gynecologists Regarding Screening of Domestic Violence against Women. BioMedica. 2021 Dec 30;37(4):253–8.

27. Ahmad A, Aziz M, Anjum G, Mir FV. Intimate Partner Violence and Psychological Distress: Mediating Role of Stockholm Syndrome. :17.

For peer review only

28. C.R. Reynolds, Kamphaus, R.W. Reynolds Intellectual Assessment Scales, Second Edition (RIAS-2). Psychol Assess Resour Inc. 2015;

29. Riaz Z, Shahzad S, Ahmad R, Khanam SJ. Psychological Adjustment among Intellectually Gifted Secondary School Children. Pak J Psychol [Internet]. 2013 Dec 31 [cited 2022 Oct 31];44(2). Available from: http://www.pjpku.com/index.php/pjp/article/view/100

30. GSED Team. The Global Scale for Early Development (GSED). Early Child Matters. 2019;14:80–4.

For peer review only

Figure legends:

Figure 1: Summary of measures collected in each of the four visits from both the mother and child.

Figure 2: The scan card of sequence and timings used for the MRI scan at all visits (1-4).

For peer review only

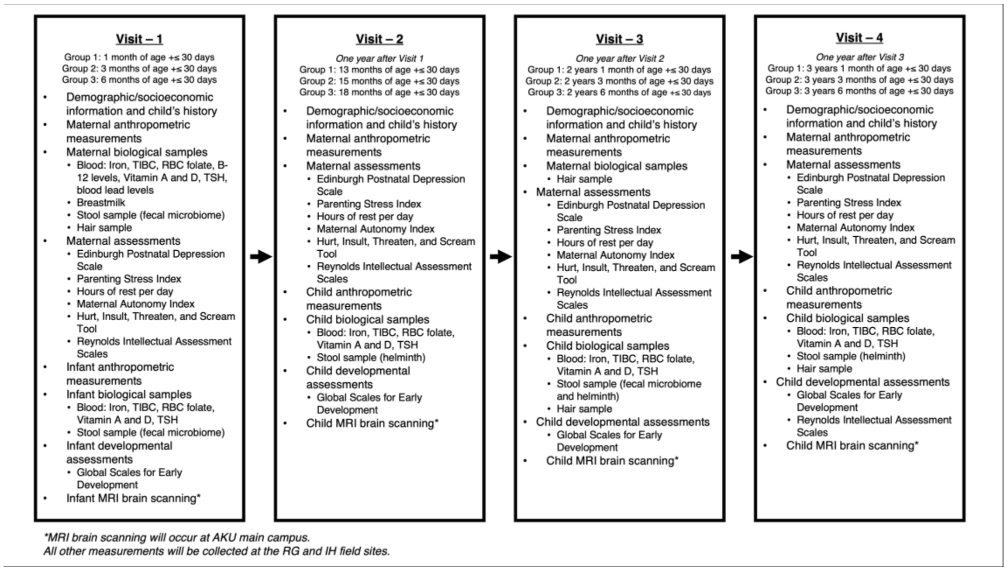

Summary of measures collected in each of the four visits from both the mother and child.

338x190mm (300 x 300 DPI)

Scan Sequence	Timing
3 Axis Locator Mapping, Shimming	00:18
CO FLAIR	02:38
DWI b1000	01:17
FSBB AX (SWI)	02:38
DWI 30 axis (DTI)	05:38
f-MRI	03:41
SAG T2 VOLUME	05:39
SG T1W (FFE3D)	05:38
3D T1wi sg +C	05:17

The scan card of sequence and timings used for the MRI scan at all visits (1-4).

133x78mm (300 x 300 DPI)

Reporting checklist for cohort study.

Based on the STROBE cohort guidelines.

Instructions to authors

Complete this checklist by entering the page numbers from your manuscript where readers will find each of the items listed below.

Your article may not currently address all the items on the checklist. Please modify your text to include the missing information. If you are certain that an item does not apply, please write "n/a" and provide a short explanation.

Upload your completed checklist as an extra file when you submit to a journal.

In your methods section, say that you used the STROBE cohortreporting guidelines, and cite them as:

von Elm E, Altman DG, Egger M, Pocock SJ, Gotzsche PC, Vandenbroucke JP. The Strengthening the Reporting of Observational Studies in Epidemiology (STROBE) Statement: guidelines for reporting observational studies.

		Reporting Item	Page Number
**Title and abstract**			
Title	#1a	Indicate the study's design with a commonly used term in the title or the abstract	1
Abstract	#1b	Provide in the abstract an informative and balanced summary of what was done and what was found	2
**Introduction**			
Background / rationale	#2	Explain the scientific background and rationale for the investigation being reported	3
Objectives	#3	State specific objectives, including any prespecified hypotheses	4
**Methods**			
Study design	#4	Present key elements of study design early in the paper	5

For peer review only

Setting	#5	Describe the setting, locations, and relevant dates, including periods of recruitment, exposure, follow-up, and data collection	4
Eligibility criteria	#6a	Give the eligibility criteria, and the sources and methods of selection of participants. Describe methods of follow-up.	5
Eligibility criteria	#6b	For matched studies, give matching criteria and number of exposed and unexposed	n/a, not completing a match study
Variables	#7	Clearly define all outcomes, exposures, predictors, potential confounders, and effect modifiers. Give diagnostic criteria, if applicable	6-8
Data sources / measurement	#8	For each variable of interest give sources of data and details of methods of assessment (measurement). Describe comparability of assessment methods if there is more than one group. Give information separately for for exposed and unexposed groups if applicable.	6-9
Bias	#9	Describe any efforts to address potential sources of bias	5
Study size	#10	Explain how the study size was arrived at	5
Quantitative variables	#11	Explain how quantitative variables were handled in the analyses. If applicable, describe which groupings were chosen, and why	6-9
Statistical methods	#12a	Describe all statistical methods, including those used to control for confounding	
9-10			
Statistical methods	#12b	Describe any methods used to examine subgroups and interactions	9-10
Statistical methods	#12c	Explain how missing data were addressed	10
Statistical methods	#12d	If applicable, explain how loss to follow-up was addressed	10
Statistical methods	#12e	Describe any sensitivity analyses	

For peer review only

n/a

Results

Participants	#13a	Report numbers of individuals at each stage of study—eg numbers potentially eligible, examined for eligibility, confirmed eligible, included in the study, completing follow-up, and analysed. Give information separately for for exposed and unexposed groups if applicable.	n/a, this is a protocol manuscript (so results are not available yet)
Participants	#13b	Give reasons for non-participation at each stage	n/a
Participants	#13c	Consider use of a flow diagram	

n/a

Descriptive data	#14a	Give characteristics of study participants (eg demographic, clinical, social) and information on exposures and potential confounders. Give information separately for exposed and unexposed groups if applicable.	n/a
Descriptive data	#14b	Indicate number of participants with missing data for each variable of interest	

n/a

Descriptive data	#14c	Summarise follow-up time (eg, average and total amount)	

n/a

Outcome data	#15	Report numbers of outcome events or summary measures over time. Give information separately for exposed and unexposed groups if applicable.	

n/a

Main results	#16a	Give unadjusted estimates and, if applicable, confounder-adjusted estimates and their precision (eg, 95% confidence interval). Make clear which confounders were adjusted for and why they were included	n/a
Main results	#16b	Report category boundaries when continuous variables were categorized	n/a
Main results	#16c	If relevant, consider translating estimates of relative risk into absolute risk for a meaningful time period	

For peer review only

n/a			
Other analyses	#17	Report other analyses done—eg analyses of subgroups and interactions, and sensitivity analyses	n/a
**Discussion**			
Key results	#18	Summarise key results with reference to study objectives	n/a, this is a protocol manuscript
Limitations	#19	Discuss limitations of the study, taking into account sources of potential bias or imprecision. Discuss both direction and magnitude of any potential bias.	n/a
Interpretation	#20	Give a cautious overall interpretation considering objectives, limitations, multiplicity of analyses, results from similar studies, and other relevant evidence.	n/a
Generalisability	#21	Discuss the generalisability (external validity) of the study results	n/a
**Other Information**			
Funding	#22	Give the source of funding and the role of the funders for the present study and, if applicable, for the original study on which the present article is based	10

The STROBE checklist is distributed under the terms of the Creative Commons Attribution License CC-BY. This checklist was completed on 12. April 2023 using https://www.goodreports.org/, a tool made by the EQUATOR Network in collaboration with Penelope.ai

For peer review only

TRIPOD Checklist: Prediction Model Development

Section/Topic	Item	Checklist Item	Page
**Title and abstract**			
Title	1	Identify the study as developing and/or validating a multivariable prediction model, the target population, and the outcome to be predicted.	1
Abstract	2	Provide a summary of objectives, study design, setting, participants, sample size, predictors, outcome, statistical analysis, results, and conclusions.	2
**Introduction**			
Background and objectives	3a	Explain the medical context (including whether diagnostic or prognostic) and rationale for developing or validating the multivariable prediction model, including references to existing models.	3
	3b	Specify the objectives, including whether the study describes the development or validation of the model or both.	4
**Methods**			
Source of data	4a	Describe the study design or source of data (e.g., randomized trial, cohort, or registry data), separately for the development and validation data sets, if applicable.	5
	4b	Specify the key study dates, including start of accrual; end of accrual; and, if applicable, end of follow-up.	5
Participants	5a	Specify key elements of the study setting (e.g., primary care, secondary care, general population) including number and location of centres.	4
	5b	Describe eligibility criteria for participants.	5
	5c	Give details of treatments received, if relevant.	n/a
Outcome	6a	Clearly define the outcome that is predicted by the prediction model, including how and when assessed.	6-9
	6b	Report any actions to blind assessment of the outcome to be predicted.	n/a
Predictors	7a	Clearly define all predictors used in developing or validating the multivariable prediction model, including how and when they were measured.	6-9
	7b	Report any actions to blind assessment of predictors for the outcome and other predictors.	n/a
Sample size	8	Explain how the study size was arrived at.	5
Missing data	9	Describe how missing data were handled (e.g., complete-case analysis, single imputation, multiple imputation) with details of any imputation method.	10
Statistical analysis methods	10a	Describe how predictors were handled in the analyses.	9-10
	10b	Specify type of model, all model-building procedures (including any predictor selection), and method for internal validation.	9-10
	10d	Specify all measures used to assess model performance and, if relevant, to compare multiple models.	n/a
Risk groups	11	Provide details on how risk groups were created, if done.	n/a
**Results**			
Participants	13a	Describe the flow of participants through the study, including the number of participants with and without the outcome and, if applicable, a summary of the follow-up time. A diagram may be helpful.	n/a
	13b	Describe the characteristics of the participants (basic demographics, clinical features, available predictors), including the number of participants with missing data for predictors and outcome.	n/a
Model development	14a	Specify the number of participants and outcome events in each analysis.	n/a
	14b	If done, report the unadjusted association between each candidate predictor and outcome.	n/a
Model specification	15a	Present the full prediction model to allow predictions for individuals (i.e., all regression coefficients, and model intercept or baseline survival at a given time point).	n/a
	15b	Explain how to the use the prediction model.	n/a
Model performance	16	Report performance measures (with CIs) for the prediction model.	n/a
**Discussion**			
Limitations	18	Discuss any limitations of the study (such as nonrepresentative sample, few events per predictor, missing data).	n/a
Interpretation	19b	Give an overall interpretation of the results, considering objectives, limitations, and results from similar studies, and other relevant evidence.	n/a
Implications	20	Discuss the potential clinical use of the model and implications for future research.	10
**Other information**			
Supplementary information	21	Provide information about the availability of supplementary resources, such as study protocol, Web calculator, and data sets.	n/a
Funding	22	Give the source of funding and the role of the funders for the present study.	10

We recommend using the TRIPOD Checklist in conjunction with the TRIPOD Explanation and Elaboration document.

For peer review only - http://bmjopen.bmj.com/site/about/guidelines.xhtml

BMJ Open

Maternal and Environmental Impact Assessment on Neurodevelopment in Early Childhood Years (MINE): A Prospective Cohort Study Protocol from a Low, Middle-Income Country

Journal:	*BMJ Open*
Manuscript ID	bmjopen-2022-070283.R2
Article Type:	Protocol
Date Submitted by the Author:	17-May-2023

Complete List of Authors:	Surani, Zoya; The Aga Khan University, Department of Paediatrics and Child Health<br>Parkar, Sadia; The Aga Khan University, Department of Paediatrics and Child Health<br>Afshan, Gul; The Aga Khan University, Department of Paediatrics and Child Health<br>Elahi, Kinza; The Aga Khan University, Department of Paediatrics and Child Health<br>Hoodbhoy, Zahra; The Aga Khan University, Department of Paediatrics and Child Health<br>Hilal, Kiran; The Aga Khan University, Department of Radiology<br>Jafri, Sidra Kaleem; The Aga Khan University, Department of Paediatrics and Child Health

**Primary Subject Heading**:	Paediatrics
Secondary Subject Heading:	Public health
Keywords:	PAEDIATRICS, Paediatric neurology < PAEDIATRICS, Community child health < PAEDIATRICS, Magnetic resonance imaging < RADIOLOGY & IMAGING, PUBLIC HEALTH

SCHOLARONE™
Manuscripts

TITLE: **M**aternal and Environmental **I**mpact Assessment on **N**eurodevelopment in **E**arly Childhood Years (MINE): A Prospective Cohort Study Protocol from a Low, Middle-Income Country

Authors: Zoya Surani[1], Sadia Parkar[1], Gul Afshan[1], Kinza Elahi[1], Zahra Hoodbhoy[1], Kiran Hilal[2], Sidra Kaleem Jafri[1]

[1]Department of Paediatrics and Child Health, The Aga Khan University, Karachi, Pakistan
[2]Department of Radiology, The Aga Khan University, Karachi, Pakistan

Corresponding author: Dr. Sidra Kaleem Jafri
Address: Stadium Road, P.O. Box 3500, Karachi 74800, Pakistan
Email address: sidra.kaleem@aku.edu

Author email addresses:
Zoya Surani (zoyasurani@gmail.com), Sadia Parkar (sadia.parkar@aku.edu), Gul Afshan (afshan.gul@aku.edu), Kinza Elahi (kinza.elahi@aku.edu), Zahra Hoodbhoy (zahra.hoodbhoy@aku.edu), Kiran Hilal (kiran.hilal@aku.edu), Sidra Kaleem Jafri (sidra.kaleem@aku.edu)

Word count: 3971

For peer review only

ABSTRACT

Introduction: Environmental and psychosocial adversities negatively impact children's developmental outcomes. When these factors are experienced in early childhood–a sensitive period of development–the developing brain can be altered. While these associations have been drawn in high-income countries, it is necessary to understand child growth, neurodevelopment, and the role of environmental factors in developmental trajectories in low-income settings. The objective of this study is to longitudinally assess how demographic factors, maternal health, maternal development, and child health are related to child development on a behavioral, cognitive, and neuroimaging level in low-socioeconomic communities.

Methods and analysis: Mother-child dyads will be identified in the peri-urban field sites of Rehri Goth and Ibrahim Hyderi, Karachi, Pakistan. Dyads will undergo yearly assessments for four years beginning when the child is 1 month, 3 months, or 6 months of age (+≤ 30 days of age) (depending on group assignment). Maternal assessments include anthropometry, behavioral, cognitive, and developmental assessments (Edinburgh Postnatal Depression Scale; Parenting Stress Index; Maternal Autonomy Index; Hurt, Insult, Threaten, Scream Tool; Reynolds Intellectual Assessment Scales), and biological samples collection (breastmilk, blood, stool, hair). Children's assessments include anthropometry, developmental assessments (Global Scales for Early Development; RIAS), MRI brain assessments, and biological sample collection (blood, stool, hair). Using cross-sectional and longitudinal data with statistical analysis tools, associations will be quantified between brain structure (magnetic resonance imaging) and connectivity (resting state connectivity and diffusion tensor imaging), general cognitive skills (RIAS, GSED), and environmental influences (nutrition via biological samples, maternal mental health via questionnaires) through repeated measures ANOVA tests and chi-square tests. Quantile regression and cortical analyses will be conducted to understand how demographic factors are related to the associations found.

Ethics and dissemination: The study has received ethical approval from the Aga Khan University Ethics Review Committee. The study's findings will be disseminated through scientific publications and project summaries for the participants.

Strength and limitations of this study:

- This is a longitudinal study that will assess various behavioral, cognitive, biological, and neuroimaging factors related to child development yearly for four years.
- There will not be a direct control group in this study.
- Though the sample size was calculated taking into account attrition that may occur over the study's timeline, a formal sample size calculation was not completed to determine the power of the study.

Keywords: paediatrics, paediatric neurology, community child health, magnetic resonance imaging, public health

For peer review only

INTRODUCTION

Worldwide, an estimated 250 million children under the age of 5 years fail to meet their developmental potential due to the exposure to early life adversities, such as malnutrition, psychosocial and environmental stress, and neglect (1). Many of these children live in low- and middle-income countries (LMICs), where there is often a profound confluence of negative environmental and psychosocial factors (2). The first 1000 days of a child's life are widely recognized as a sensitive period of development, where the structural and functional architecture of the brain is laid, and lifelong patterns of physical, cognitive, and socio-emotional health are established (3).

Throughout this period, the fetal and infant's brain increases almost three-fold in volume, and its extensive neuroarchitecture matures through processes including neurogenesis, migration, synaptogenesis, and myelination (4). This transformation is fueled by the mother and child's nutritional and energy resources, and shaped by cascades of genetic and environmental interactions–modulated through psychosocial and caregiving relationships (3,5). Previous neuroimaging studies provide clear evidence of how these factors can alter the developing brain, particularly when these adversities are experienced during the early life years (6).

Infants are inherently competent in their ability to initiate relationships, explore, seek meaning, and learn, but are also vulnerable and depend entirely on caregivers for their survival, emotional security, modeling of behaviors, and to teach them the norms of the physical and socio-cultural world that they inhabit (7). This sensitive period, then, is a double-edged sword. While positive and enriching environments can promote healthy brain development, neglect and abuse can impair maturing brain systems and disrupt cognitive and behavioral outcomes (8,9). Early adversities disrupt normative developmental trajectories with lifelong consequences, including impaired intellectual functioning, depression, and other mental health disorders (6,10). Therefore, adversity and resource inequality not only contribute to poor brain development but are also an outcome of it, potentially driving a vicious intergenerational cycle (11).

While existing literature on this topic has largely focused on understanding the neurodevelopmental impact of environmental factors as they exist in high-income areas, literature is evolving to include more data from LMICs–as understanding the nature of adversities in different environments is an important consideration, particularly in LMICs where the burden to do so is greater. Of the studies that aim to understand neurodevelopment, there has been a recent focus on using magnetic resonance imaging (MRI) as the preferred modality of neuroimaging due to its anatomical tissue contract, ability to visualize anatomical structure and physiological function, and its safety in use (12). By using MRI to image young children, there is a greater ability to visualize and understand brain plasticity, and development of perceptual and cognitive skills (13).

The two major studies conducted on this topic in LMICs were completed in Bangladesh and India. In the Bangladesh Early Adversity Neuroimaging study, associations between psychosocial risks (maternal distress, caregiving experiences), biological risks (growth, inflammation), developmental outcomes (Mullen Scales, Wechsler Preschool and Primary Scale, Bayley Scales), and neurodevelopment (electroencephalography, MRI) were assessed in children aged 6 months to 7 years (14). In this study, as well as another conducted in India to understand

For peer review only

neurodevelopmental outcomes in infants up to 18 months of age, researchers found that adverse factors present in LMIC environments are associated with outcomes on the cognitive and neural level (14,15). These studies highlight the importance of using MRI in LMICs to investigate the impact of these environments.

Despite the presence of studies in LMICs aimed at explaining children's neurodevelopment in these environments, there is a decreased understanding of the mechanisms involved in how and why children's outcomes are impacted. These are important considerations because before effective interventions can be designed to minimize adversities and their impact on children's development and neurodevelopment, it is necessary to understand the role of environmental factors. Knowing that the early years are a time of immense brain development and extremely susceptible to factors in the environment, it is necessary to comprehensively map children's growth and development–assessing both environmental factors and brain structure continuously in low-resource environments.

Pakistan is among the countries with the highest rates of malnutrition and its progress in perinatal, neonatal, and child health has been much slower than in other South Asian countries (16,17). Karachi, the largest metropolitan city in Pakistan and 12th largest city in the world with a linguistically, ethnically, and religiously diverse population is an excellent surrogate to study the impact of environment on a child's development. The primary objective of the current study is to longitudinally map trajectories of child growth and neurodevelopment from early infancy (1 month) to 4 years of age using longitudinal MRI scanning and neurodevelopmental assessments in Karachi, Pakistan. The secondary objective of this study is to investigate the relationships between modifiable environmental factors (e.g., early child nutrition, maternal stress) with evolving brain structure, brain function, and maturing cognitive skills. We hypothesize that children with exposure to conditions like maternal and child malnutrition and adverse childhood experiences will have poor development with macro-level structural brain differences in comparison to children who were not exposed to these conditions.

METHODS AND ANALYSIS

Setting

In Karachi, to facilitate the large number of patients per day, The Aga Khan University Hospital has created a massive network with 4 secondary care hospitals, 30+ medical centers, 290+ clinical laboratories, and 20+ pharmacies in over 120 cities, and also provides home health care services across Pakistan. Aga Khan University Hospital in Karachi is connected with many primary health care clinics (PHCs) in the peri-urban coastal regions of the city and has a 3T MRI machine available onsite. The PHCs are maintained by the Department of Paediatrics and Child Health at AKU, and have quarterly household surveillance for key maternal and child health indicators along with antenatal care, immunisation and physician services for children under the age of 5 years. For these reasons, the provision of services at PHCs connected with the larger tertiary hospital make this area an ideal setting for this study. The study will be conducted at two of these PHCs, Rehri Goth (RG) and Ibrahim Hyderi (IH), which have been operating in the communities for over a decade.

Design

For peer review only

This study is a longitudinal cohort study and will be built upon the Pregnancy Risk Stratification Innovation and Measurement Alliance (PRiSMA) study (ERC Number: 2021-5920-15518). The PRiSMA study is a longitudinal study that enrolls pregnant women within the first 20 weeks of gestation and follows them and their newborns until one year after delivery. The current study will map trajectories of child growth and neurodevelopment from 1 month to 3.5 years of age. Additionally, the reporting of this study conforms to the STROBE statement (18).

Duration

The study will be conducted over a 3.5-year period (May 2022 through December 2026).

Inclusion and exclusion criteria

The inclusion criteria are:

- Singleton infants aged 1 month +≤ 30 days of age, 3 months +≤ 30 days of age, or 6 months +≤ 30 days of age of age and their mothers

The exclusion criteria are:

- Infants with congenital anomalies (established on clinical visits)
- Infants with history of hypoxic-ischemic insult, delayed cry, requiring oxygen, or hospitalization
- Maternal usage of anti-depressive or anti-epileptic medications during pregnancy
- Fetal exposure to maternal smoking (cigarettes)
- Ultrasound abnormalities (e.g., ventriculomegaly, renal agenesis, gastrointestinal structural anomalies, etc.)
- Conditions like preeclampsia, high blood pressure, or diabetes mellitus in pregnancy

Sample size calculation

A formal sample size calculation to obtain the power of the study was not calculated. We aimed to collect data on 150 mother-child dyad pairs over the study duration, basing our sample size on what was found feasible in previous neuroimaging infant studies (14,15). However, keeping attrition of 40% due to identified challenges (e.g., difficulty to obtain follow-up data due to family relocation, family refusal, etc.), approximately 250 mother-child dyads will be recruited.

Recruitment procedure

A list of households with newborns will be shared by the PRiSMA study team on a monthly basis. After households with infants of appropriate age are identified, mother-child dyads will be approached via simple random sampling to reduce any sampling bias. At the mother's home, research staff will ask questions related to their and their child's medical history to determine their eligibility (based on the exclusion criteria).

Once written informed consent is provided by the parent/guardian, the enrollment visit will be performed, and a unique study participant identification number (ID) will be assigned to the infant and mother, where they will both have the same ID number, but labeled with "infant" or "mother" to distinguish them (e.g., infant ID: Infant-1234, mother ID: Mother-1234). The participants will be recruited in three groups: Group 1 will include 100 mother-infant dyads with infants that are 1 month +≤ 30 days of age, Group 2 will include 100 mother-infant dyads with infants that are 3 months +≤ 30 days of age, and Group 3 will include 50 mother-infant dyads with infants that are 6 months +≤ 30 days of age. As neuroimaging scans in natural sleep are more likely to be

For peer review only

successful in infants of younger age and MRI scans with sedation may be more challenging to obtain enrollments for, the group sizes of the younger groups (1-month and 3-month) are larger than that of the older infants (6-months). This split will also allow the team to obtain images and samples at different timepoints during the early childhood period without the need for repetitive sampling of any one mother-child dyad.

The study involves the mother-child dyads completing four yearly visits, beginning when the child is an infant (1 month/3 months/6 months) until they are 3 years and 1 month/3 months/6 months of age. Each visit will comprise of the child's MRI brain scan, followed by the collection of information and biological samples. For the MRI scan, the family will be transported to AKU, accompanied by members of the research team. Once the scan is completed, estimated to take no longer than 1 hour (prescanning (5 minutes), child placement (10 minutes), scanning time (30+ minutes, depending on repeated measures)), the demographic information, maternal physical and mental health assessments, the child's history, child physical health and developmental assessments, and biological samples will be collected on the following day at the PHC at the study site. The collection of the assessments and samples will take approximately two hours. The collection procedure of non-MRI data will be adapted to ensure effective use of resources once the MRI scan is successfully performed. Additionally, quality control programs are in place for data collection to ensure there are no changes in the measurement of variables over the study's duration. Weekly quality checks will be conducted under the supervision of master trainers for all behavioral, cognitive, and developmental assessment tools used in the study. For the neuroimaging data, to ensure the scanner is operating correctly and producing reliable images, radiologists conduct routine quality control and assurance procedures weekly, such as system checks and calibrations (with a phantom), under the supervision of the biomedical team. In addition, a service engineer performs thorough diagnostic/maintenance tests on a quarterly basis to confirm the machine is working as intended. If there are any machine or software malfunctions, scans will not be completed during that time. Lastly, the quality of biological samples will be checked by the lab team and frequent quality management of all collected data will be done by the Data Management Unit.

A summary of the visits is provided in Figure 1 and descriptions of each measure to be collected are provided in the next section.

Measures of outcomes

1) Demographics and history
    - *Demographic/socioeconomic information*
      Demographic and socioeconomic data will be collected at all visits (1-4). The data will include detailed maternal pregnancy history (gravidity, live births, pregnancy spacing, maternal age at each pregnancy), family composition (child ages and sex), socioeconomic and household asset information (mother and father education and occupation, religion, caste, household income, home size and number of rooms, toilet type, electricity access, number of mobile phones, housing type, cooking fuel, home, land and/or animal ownership, and transportation access).
    - *Child's history*
      A detailed assessment regarding the child's well-being, co-morbidities if any, major hospitalizations, child diarrhea events, antibiotic use, and vaccination history will be

For peer review only

collected at all visits. Labor and delivery information, as well as birth anthropometry, will be obtained from the PRiSMA records.

2) Maternal assessments

- *Physical health assessments*
  i. Anthropometric measurements: Assessment of maternal anthropometry i.e. weight and height will be recorded using a calibrated weighing scale (Seca 874 Mobile Flat Scale) and measuring tape (Seca 212 Measuring Tape), respectively, at all visits.
  ii. Biological sample collection: Lab investigations of serum iron, total iron-binding capacity (TIBC), red blood cell (RBC) folate, Vitamin B-12 levels, Vitamins A and D, blood lead levels, and thyroid stimulating hormone (TSH) will be done at visit 1. A sample of the mother's hair will be collected at visit 1 and 3 to assess for cortisol. A breastmilk sample (20 mL) will be collected from the mother at visit 1 and the composition (general macronutrient (protein, fat, and carbohydrate content), as well as cholesterol, choline, and vitamin B content) will be determined. Stool sample for fecal microbiome analysis will be collected from the mother at visit 1.
- *Behavioral and cognitive assessments*
  Behavioral and cognitive assessments will be administered at all visits.
  i. Maternal mental health will be assessed by using Urdu translations of the Edinburgh Postnatal Depression Scale (EPDS) (19), Parenting Stress Index (PSI) long-form (20), and through collecting the hours of maternal rest per day. The EPDS and PSI have been locally validated (21–23).
  ii. Maternal autonomy, family violence, addiction, and neglect will be assessed using Urdu-translated versions of the Maternal Autonomy Index (24), and the Hurt, Insult, Threaten, and Scream (HITS) Tool (25), which have been locally validated (26,27).
- *Developmental assessment*
  The Reynolds Intellectual Assessment Scales (RIAS) (28) will be used to assess the mother's development at all visits. The RIAS measures general intelligence and nonverbal intelligence, and is administered to individuals ages 3 to 94. The RIAS has been locally validated (29).

3) Child assessments

- *Physical health assessments*
  i. Anthropometric measurements: Weight, height, and head circumference of the child will be recorded using a calibrated weighing scale (Seca 334 Mobile Digital Baby Scale), infant measuring board (Seca 416 Infantometer), and measuring tape (Seca 212 Measuring Tape), respectively. Mid-upper arm circumference will also be measured for children above six months of age at every visit. The WHO Z-scores will be used for anthropometric interpretation and standards for weight, length/height, weight-for-length/height, and head circumference-for-age will be calculated. Child anthropometric measurements will be collected yearly (visits 1-4) and analyzed within their enrollment group and visit number.

For peer review only

ii. Biological sample collection: Lab investigations of serum iron, TIBC, RBC folate, Vitamin B-12 levels, Vitamins A and D, blood lead levels, and TSH will be completed yearly (visits 1-4). Stool samples will be collected yearly (visits 1-4) and will be assessed for fecal microbiome (visits 1 and 3) and helminth (visits 2, 3, and 4). Hair sample to assess cortisol levels will be collected at visits 3 and 4.

- *Developmental assessments*

The Global Scales for Early Development (GSED) (30) (0 to 6 years) and Reynolds Intellectual Assessment Scales (RIAS) (non-verbal) (3 to 94 years) tools will be used to assess the child's development. The GSED will be administered at all visits (1-4), while the RIAS will only be administered to the child at visit 4 (when the child is >3 years old).

i. GSED: The World Health Organization (WHO) has developed and is currently validating the GSED, which can be used to assess children's development in the early years of life across the globe, including in Pakistan. As part of this scale, the short form (SF) and long-form (LF) will be used. Both SF and LF are unidimensional and provide a single age-adjusted score that represents a child's level of development: The SF uses a caregiver-reported questionnaire while the LF is a directly administered (observed) tool assessing the child's abilities and behaviors.

ii. RIAS: The RIAS measures general intelligence and nonverbal intelligence, and is administered to individuals ages 3 to 94. As the participants will be >3 years old only at visit 4, the RIAS will be administered only at that visit.

- *MRI brain scanning*

At every visit (1-4), MRI scans (lasting no longer than one hour) will be conducted using a 3T MRI machine (Toshiba Titan, Canon Medical Systems) equipped with a 32-channel coil, which provides high field strength, multi-phase imaging, and advanced motion correction capabilities. To optimize the functionality and performance of the Titan scanner, the specialized Ventage 2.5 software will be utilized.

The sequences that will be performed as part of analysis for this study include diffusion tensor imaging, T1-Weighted MPRAGE and T2 Volume anatomical imaging, and functional MRI. Information about the key parameters and sequence timing are included in Table 1. As part of the hospital protocol, Fluid attenuated inversion recovery (FLAIR) and Flow Sensitive Black Blood (FSBB) susceptibility-weighted imaging (SWI) will also be performed and reviewed by a physician, but these sequences will solely be used for clinical information. The parameters for these sequences are included in Supplementary Information, Table 1.

Table 1: Key parameters of MRI sequences performed at all visits

Sequence	***Diffusion Weighted Imaging 30 axis (DTI)***	***Functional MRI***	***T2 Volume***	***3D T1-Weighted MPRAGE***
Orientation	Axial	Axial	Sagittal	Sagittal
TR (ms)	5090	2400	3200	1819.5
TE (ms)	82	25	352	3.384
FA (degrees)	90	90	90	12

For peer review only

FOV (mm)	230x230	240x240	231x231	160x160
Matrix size	160x160	96x96	512x512	512x512
#Slices	28	21	260	150
Thickness (mm)	4	3	1	1.48
Spacing (mm)	4	4	0.5	0.75
Voxel size (mm^3)	1.438x 1.438x4	2.5x 2.5x4	0.451x 0.451x0.5	0.312x 0.312x0.75
Phase Encoding Direction	j; Anterior to Posterior (A to P)	j; A to P	i; Right to Left (R to L)	i; R to L
b-values (sec/mm^2)	0/1000			
Time (s)	05:38	03:41	04:32	09:55

For MRI brain scanning, the mother and child will be brought to AKU accompanied with members of the research team. Once the child appears to be in deep sleep (15-20 mins after the child went to sleep), the MRI will be performed. The child will then be secured in an immobilizer (MedVac Immobilization Bag, Infant Splint; MRI Med, Petaluma, CA, USA) and transferred from their crib or bed to an MRI-compatible cart. The immobilizer will be used to reduce subtle body movement during the scan (e.g., movement from deep sleep breathing). The child's head will be carefully positioned, and a removable sound-insulating foam insert (Ultra Barrier HD Composite; American Micro Industries, Chambersburg, PA, USA) will be securely placed on the child's ears. The process will be facilitated by a research coordinator, and parents will be invited to stay with the infant to allay any anxiety. A pediatric pulse oximeter will be attached to the child's finger or toe to monitor the child during the scan. A research assistant will remain inside the scanning suite in case the child wakes up during the scan.

With children scanned in natural sleep, there is a possibility that they may wake up during the scan. If this happens, the child will be removed from the scanner and nursed and swaddled prior to continuing the scan. The sequences affected by participant motion will be repeated once the child is asleep again.

The scan will ideally be performed without sedation at any stage of the scan, using strategies developed by Dean et al (13). In case the child does not sleep, we will use minimally conscious sedation routinely employed in the pediatric population. For infants up to 3 months this includes chloral hydrate, 25mg per kg given orally, while for children 6 months and above, procedural sedation will be used which will be carried out by a credentialed physician and sedation nurse. The sedation medication will be titrated according to the child's weight and children requiring sedation will be admitted for pre, intra, and post sedation monitoring.

During the follow-up visits, though the imaging data may be difficult to acquire in unsedated toddlers, the scans will be initially attempted in natural sleep. The data will be carefully reviewed to exclude low quality data and data that exceeds our motion threshold. Scans with sedation will be used as a last resort and will be performed rarely due to possible risks that might occur with annual evaluations with sedation.

Patient and public involvement

Patients and the public were not involved in the design of this study. However, study participants will be informed regarding the study findings after the study results are published and their help will be enlisted to help with the dissemination of research findings.

For peer review only - http://bmjopen.bmj.com/site/about/guidelines.xhtml

Statistical analysis plan

Analysis of participant characteristics will be conducted using STATA version 17. Participant characteristics will be described using means and standard deviations for continuous variables (anthropometric measurements, maternal behavioral, cognitive, and developmental assessments scoring, and child developmental assessments scoring) and percentages for categorical variables (biological samples, demographics, and child's history information).

MRI brain analysis will be performed locally, as experts from the funding agency will train the local Data Management Unit team at AKU. MRI data will be quality checked by a radiologist and radiologist technician to ensure there were no abnormalities found, all sequences were completed, and there was limited motion. Visualization of data will be done using Mango and Horos tools. Analysis of neuroimaging data will be completed using the Flywheel platform and the embedded FreeSurfer, FSL, and MRtrix3 analysis tools. Data will be segmented, skull-stripped, scaled to standard space, and registered to age-matched templates that will be created locally and matched with existing templates (e.g., NIH). For structural data, estimates of total brain tissue volume, thickness, and surface area will be acquired. Functional MRI data will be preprocessed and motion corrected to analyze differences in resting-state activity (as the scans will occur in natural or sedated sleep). Fractional anisotropy values will be obtained from the diffusion imaging to understand the shape of diffusion and will be visualized with tractography.

Ultimately, patterns of structural and functional development will be characterized, and associations will be conducted between cognitive outcomes and deviations associated with birth outcomes and environmental factors at various time points. Imaging data will be paired with neurocognitive assessments (GSED, RIAS, etc.) performed at predetermined intervals (GSED at visits 1-3, RIAS at visit 4). We will quantify the associations (e.g., with a chi-square test) between maternal and child health (via levels of micronutrients in the blood and breastmilk) on measures of fetal and infant growth and neurodevelopment. We will use repeated measures ANOVA tests to determine statistically significant differences between measures collected at various time points–child anthropometric measurements, biological samples, child developmental assessment scores, and child MRI scanning via volume estimates through structural MRI, resting-state connectivity patterns through fMRI, and functional connectivity through DTI. Quantile regression analyses will be performed to understand if demographic factors (e.g., socioeconomic status) and/or child sex are related to any of the associations found between cognitive skills, environmental influences, and emerging neurobehavior. Cortical thickness analyses will be conducted to specifically understand the correlation between demographic factors and structural differences (via cortical thickness as found through T1W MRI).

As this is a longitudinal study, due to identified challenges that may result in a loss of follow-up, we have kept attrition at 40%, thus increasing our participant recruitment to 250 mother-child dyads for our ultimate sample size of 150 mother-child dyads. Additionally, during the analysis, if there is missing data, the data will be addressed appropriately to ensure the remaining data are able to be maximally used.

ETHICS AND DISSEMINATION

Ethics approval of the study was obtained from the Ethics Review Committee of Aga Khan University (Reference Number: 2022-7181-21831). Written informed consent will be voluntarily

For peer review only

obtained from all participants, and participants will be informed of their complete right of refusal to initiate or withdraw at any stage of the study with no obligations. Consent for the biological samples will be obtained as part of the consent for participation in the study. Hospital data transfer policy will be employed, and all data collected and/or shared from participants will be de-identified.

The study's findings will be disseminated through scientific publications and medical education seminars. Participants will be informed of the study's findings through project summaries with the key findings included. As this study may provide insight to factors in the environment that will influence child development, the key points from the study will be shared with stakeholders and policymakers (e.g., Pakistan Pediatrics Association, Government of Sindh), who can use the study's findings to plan interventions for the physical and mental health of mothers and children at national, regional and international levels.

Acknowledgments: We thank our Aga Khan University Senior Research Assistants Sahiba Inayat, Adeeba Samreen, Shumaila Samito, our data collectors, and field team for their help in project management and development.

Author Contributions: ZS, SP, GA, KE, KH, ZH and SK were involved in the concept, grant writing protocol development and intellectual inputs for this project. SK, ZH and KH provided expert advice and technical training. All authors contributed towards development of this manuscript.

Funding: This work was supported by The Bill & Melinda Gates Foundation (grant number: INV-004939).

Competing interests: None declared.

License Agreement: I, the Submitting Author Zoya Surani, has the right to grant and does grant on behalf of all authors of the Work (as defined in the below author licence), an exclusive licence and/or a non-exclusive licence for contributions from authors who are: i) UK Crown employees; ii) where BMJ has agreed a CC-BY licence shall apply, and/or iii) in accordance with the terms applicable for US Federal Government officers or employees acting as part of their official duties; on a worldwide, perpetual, irrevocable, royalty-free basis to BMJ Publishing Group Ltd ("BMJ") its licensees and where the relevant Journal is co-owned by BMJ to the co-owners of the Journal, to publish the Work in BMJ Open and any other BMJ products and to exploit all rights, as set out in our licence.

The Submitting Author accepts and understands that any supply made under these terms is made by BMJ to the Submitting Author unless you are acting as an employee on behalf of your employer or a postgraduate student of an affiliated institution which is paying any applicable article publishing charge ("APC") for Open Access articles. Where the Submitting Author wishes to make the Work available on an Open Access basis (and intends to pay the relevant APC), the terms of reuse of such Open Access shall be governed by a Creative Commons licence – details of these licences and which Creative Commons licence will apply to this Work are set out in our licence referred to above.

REFERENCES

1. Black MM, Walker SP, Fernald LCH, Andersen CT, DiGirolamo AM, Lu C, et al. Early childhood development coming of age: science through the life course. The Lancet. 2017 Jan 7;389(10064):77–90.

2. Johnson SB, Riis JL, Noble KG. State of the Art Review: Poverty and the Developing Brain. Pediatrics. 2016 Apr;137(4):e20153075.

3. Likhar A, Patil MS. Importance of Maternal Nutrition in the First 1,000 Days of Life and Its Effects on Child Development: A Narrative Review. Cureus. 2022;14(10):e30083.

4. Houston SM, Herting MM, Sowell ER. The Neurobiology of Childhood Structural Brain Development: Conception Through Adulthood. Curr Top Behav Neurosci. 2014;16:3–17.

5. Stiles J, Jernigan TL. The basics of brain development. Neuropsychol Rev. 2010 Dec;20(4):327–48.

6. Nelson CA, Gabard-Durnam LJ. Early Adversity and Critical Periods: Neurodevelopmental Consequences of Violating the Expectable Environment. Trends Neurosci. 2020 Mar;43(3):133–43.

7. Köster M, Kayhan E, Langeloh M, Hoehl S. Making Sense of the World: Infant Learning From a Predictive Processing Perspective. Perspect Psychol Sci. 2020 May;15(3):562–71.

8. Ilyka D, Johnson MH, Lloyd-Fox S. Infant social interactions and brain development: A systematic review. Neurosci Biobehav Rev. 2021 Nov 1;130:448–69.

9. Miguel PM, Pereira LO, Silveira PP, Meaney MJ. Early environmental influences on the development of children's brain structure and function. Dev Med Child Neurol. 2019;61(10):1127–33.

10. Bick J, Nelson CA. Early Adverse Experiences and the Developing Brain. Neuropsychopharmacol Off Publ Am Coll Neuropsychopharmacol. 2016 Jan;41(1):177–96.

11. Walker SP, Wachs TD, Grantham-McGregor S, Black MM, Nelson CA, Huffman SL, et al. Inequality in early childhood: risk and protective factors for early child development. Lancet Lond Engl. 2011 Oct 1;378(9799):1325–38.

12. Copeland A, Silver E, Korja R, Lehtola SJ, Merisaari H, Saukko E, et al. Infant and Child MRI: A Review of Scanning Procedures. Front Neurosci. 2021;15. Available from: https://www.frontiersin.org/articles/10.3389/fnins.2021.666020

13. Dean DC, Dirks H, O'Muircheartaigh J, Walker L, Jerskey BA, Lehman K, et al. Pediatric neuroimaging using magnetic resonance imaging during non-sedated sleep. Pediatr Radiol. 2014 Jan;44(1):64–72.

14. Jensen SKG, Kumar S, Xie W, Tofail F, Haque R, Petri WA, et al. Neural correlates of early adversity among Bangladeshi infants. Sci Rep. 2019 Mar 5;9(1):3507.

For peer review only

15. Aker K, Thomas N, Adde L, Koshy B, Martinez-Biarge M, Nakken I, et al. Prediction of outcome from MRI and general movements assessment after hypoxic-ischaemic encephalopathy in low-income and middle-income countries: data from a randomised controlled trial. Arch Dis Child - Fetal Neonatal Ed. 2022 Jan;107(1):32–8.

16. Bhutta ZA, Hafeez A, Rizvi A, Ali N, Khan A, Ahmad F, et al. Reproductive, maternal, newborn, and child health in Pakistan: challenges and opportunities. Lancet Lond Engl. 2013 Jun 22;381(9884):2207–18.

17. Khan GN, Turab A, Khan MI, Rizvi A, Shaheen F, Ullah A, et al. Prevalence and associated factors of malnutrition among children under-five years in Sindh, Pakistan: a cross-sectional study. BMC Nutr. 2016 Nov 24;2(1):69.

18. von Elm E, Altman DG, Egger M, Pocock SJ, Gøtzsche PC, Vandenbroucke JP. The Strengthening the Reporting of Observational Studies in Epidemiology (STROBE) Statement: Guidelines for Reporting Observational Studies. Ann Intern Med. 2007 Oct 16;147(8):573–7.

19. Cox JL, Holden JM, Sagovsky R. Detection of postnatal depression. Development of the 10-item Edinburgh Postnatal Depression Scale. Br J Psychiatry J Ment Sci. 1987 Jun;150:782–6.

20. Loyd BH, Abidin RR. Revision of the Parenting Stress Index. J Pediatr Psychol. 1985 Jun 1;10(2):169–77.

21. Humayun A, Haider II, Imran N, Iqbal H, Humayun N. Antenatal depression and its predictors in Lahore, Pakistan. East Mediterr Health J. 2013 Apr 1;19(04):327–32.

22. Atif M, Halaki M, Chow CM, Raynes-Greenow C. Risk factors of paternal postnatal depression in Pakistan: Findings from an urban sample. Nurs Health Sci. 2022;24(3):618–24.

23. Ahmad S, Khanam SJ. Parenting Stress Related to Behavior Problems of Children with Hearing Impairment. J Pak Psychiatr Soc. 2015 Jan;12(1):51–2.

24. IndiKit. Women's Autonomy Index [Internet]. 2021 [cited 2022 Oct 20]. Available from: https://www.indikit.net/indicator/327-women-s-autonomy-index

25. Sherin KM, Sinacore JM, Li XQ, Zitter RE, Shakil A. Hits: A short domestic violence screening tool for use in a family practice setting. Fam Med. 1998;30(7):508–12.

26. Nafees R, Safdar Z, Baig FS, Sher SJ. Knowledge and Perception Among Gynecologists Regarding Screening of Domestic Violence against Women. BioMedica. 2021 Dec 30;37(4):253–8.

27. Ahmad A, Aziz M, Anjum G, Mir FV. Intimate Partner Violence and Psychological Distress: Mediating Role of Stockholm Syndrome. :17.

For peer review only

28. C.R. Reynolds, Kamphaus, R.W. Reynolds Intellectual Assessment Scales, Second Edition (RIAS-2). Psychol Assess Resour Inc. 2015;

29. Riaz Z, Shahzad S, Ahmad R, Khanam SJ. Psychological Adjustment among Intellectually Gifted Secondary School Children. Pak J Psychol [Internet]. 2013 Dec 31 [cited 2022 Oct 31];44(2). Available from: http://www.pjpku.com/index.php/pjp/article/view/100

30. GSED Team. The Global Scale for Early Development (GSED). Early Child Matters. 2019;14:80–4.

For peer review only

Figure 1: Summary of measures collected in each of the four visits from both the mother and child.

For peer review only

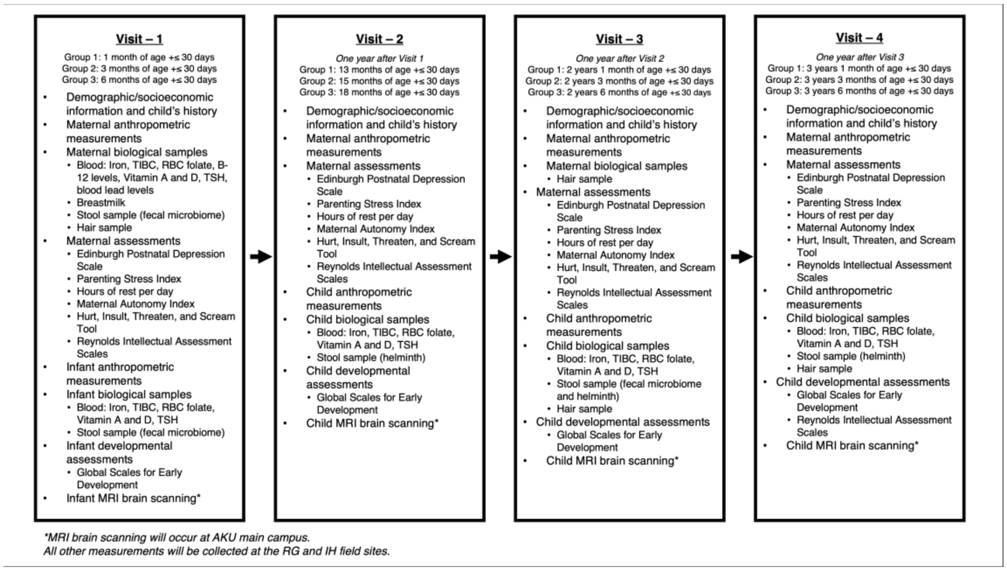

Summary of measures collected in each of the four visits from both the mother and child.

338x190mm (330 x 330 DPI)

Supplementary Information

Table 1: Parameters for FLAIR and SWI sequences

Sequence	***FLAIR***	***FSBB SWI (1)***	***FSBB SWI (2)***
Orientation	Coronal	Axial	Axial
TR (ms)	10000	29	29
TE (ms)	120	20	20
FA (degrees)	90	10	10
FOV (mm)	200x200	220x220	220x220
Matrix size	640x640	576x576	576x576
#Slices	24	9	104
Thickness (mm)	4	20	2
Spacing (mm)	5	1	1
Voxel size (mm^3)	0.312x 0.312x5	0.382x 0.382x10	0.382x 0.382x1
Phase Encoding Direction	i; R to L	j; A to P	j; A to P
Time (s)	02:20	02:10	02:10

For peer review only

Reporting checklist for cohort study.

Based on the STROBE cohort guidelines.

Instructions to authors

Complete this checklist by entering the page numbers from your manuscript where readers will find each of the items listed below.

Your article may not currently address all the items on the checklist. Please modify your text to include the missing information. If you are certain that an item does not apply, please write "n/a" and provide a short explanation.

Upload your completed checklist as an extra file when you submit to a journal.

In your methods section, say that you used the STROBE cohortreporting guidelines, and cite them as:

von Elm E, Altman DG, Egger M, Pocock SJ, Gotzsche PC, Vandenbroucke JP. The Strengthening the Reporting of Observational Studies in Epidemiology (STROBE) Statement: guidelines for reporting observational studies.

		Reporting Item	Page Number
**Title and abstract**			
Title	#1a	Indicate the study's design with a commonly used term in the title or the abstract	1
Abstract	#1b	Provide in the abstract an informative and balanced summary of what was done and what was found	2
**Introduction**			
Background / rationale	#2	Explain the scientific background and rationale for the investigation being reported	3
Objectives	#3	State specific objectives, including any prespecified hypotheses	4
**Methods**			
Study design	#4	Present key elements of study design early in the paper	5

For peer review only

Setting	#5	Describe the setting, locations, and relevant dates, including periods of recruitment, exposure, follow-up, and data collection	4
Eligibility criteria	#6a	Give the eligibility criteria, and the sources and methods of selection of participants. Describe methods of follow-up.	5
Eligibility criteria	#6b	For matched studies, give matching criteria and number of exposed and unexposed	n/a, not completing a match study
Variables	#7	Clearly define all outcomes, exposures, predictors, potential confounders, and effect modifiers. Give diagnostic criteria, if applicable	6-8
Data sources / measurement	#8	For each variable of interest give sources of data and details of methods of assessment (measurement). Describe comparability of assessment methods if there is more than one group. Give information separately for for exposed and unexposed groups if applicable.	6-9
Bias	#9	Describe any efforts to address potential sources of bias	5
Study size	#10	Explain how the study size was arrived at	5
Quantitative variables	#11	Explain how quantitative variables were handled in the analyses. If applicable, describe which groupings were chosen, and why	6-9
Statistical methods	#12a	Describe all statistical methods, including those used to control for confounding	
10			
Statistical methods	#12b	Describe any methods used to examine subgroups and interactions	10
Statistical methods	#12c	Explain how missing data were addressed	10
Statistical methods	#12d	If applicable, explain how loss to follow-up was addressed	10
Statistical methods	#12e	Describe any sensitivity analyses	

For peer review only

n/a

Results

Participants	#13a	Report numbers of individuals at each stage of study—eg numbers potentially eligible, examined for eligibility, confirmed eligible, included in the study, completing follow-up, and analysed. Give information separately for for exposed and unexposed groups if applicable.	n/a, this is a protocol manuscript (so results are not available yet)
Participants	#13b	Give reasons for non-participation at each stage	n/a
Participants	#13c	Consider use of a flow diagram	

n/a

Descriptive data	#14a	Give characteristics of study participants (eg demographic, clinical, social) and information on exposures and potential confounders. Give information separately for exposed and unexposed groups if applicable.	n/a
Descriptive data	#14b	Indicate number of participants with missing data for each variable of interest	

n/a

Descriptive data	#14c	Summarise follow-up time (eg, average and total amount)	

n/a

Outcome data	#15	Report numbers of outcome events or summary measures over time. Give information separately for exposed and unexposed groups if applicable.	

n/a

Main results	#16a	Give unadjusted estimates and, if applicable, confounder-adjusted estimates and their precision (eg, 95% confidence interval). Make clear which confounders were adjusted for and why they were included	n/a
Main results	#16b	Report category boundaries when continuous variables were categorized	n/a
Main results	#16c	If relevant, consider translating estimates of relative risk into absolute risk for a meaningful time period	

For peer review only

n/a			
Other analyses	#17	Report other analyses done—eg analyses of subgroups and interactions, and sensitivity analyses	n/a
**Discussion**			
Key results	#18	Summarise key results with reference to study objectives	n/a, this is a protocol manuscript
Limitations	#19	Discuss limitations of the study, taking into account sources of potential bias or imprecision. Discuss both direction and magnitude of any potential bias.	n/a
Interpretation	#20	Give a cautious overall interpretation considering objectives, limitations, multiplicity of analyses, results from similar studies, and other relevant evidence.	n/a
Generalisability	#21	Discuss the generalisability (external validity) of the study results	n/a
**Other Information**			
Funding	#22	Give the source of funding and the role of the funders for the present study and, if applicable, for the original study on which the present article is based	11

The STROBE checklist is distributed under the terms of the Creative Commons Attribution License CC-BY. This checklist was completed on 17. May 2023 using https://www.goodreports.org/, a tool made by the EQUATOR Network in collaboration with Penelope.ai

For peer review only

TRIPOD Checklist: Prediction Model Development

Section/Topic	Item	Checklist Item	Page
**Title and abstract**			
Title	1	Identify the study as developing and/or validating a multivariable prediction model, the target population, and the outcome to be predicted.	1
Abstract	2	Provide a summary of objectives, study design, setting, participants, sample size, predictors, outcome, statistical analysis, results, and conclusions.	2
**Introduction**			
Background and objectives	3a	Explain the medical context (including whether diagnostic or prognostic) and rationale for developing or validating the multivariable prediction model, including references to existing models.	3
	3b	Specify the objectives, including whether the study describes the development or validation of the model or both.	4
**Methods**			
Source of data	4a	Describe the study design or source of data (e.g., randomized trial, cohort, or registry data), separately for the development and validation data sets, if applicable.	5
	4b	Specify the key study dates, including start of accrual; end of accrual; and, if applicable, end of follow-up.	5
Participants	5a	Specify key elements of the study setting (e.g., primary care, secondary care, general population) including number and location of centres.	4
	5b	Describe eligibility criteria for participants.	5
	5c	Give details of treatments received, if relevant.	n/a
Outcome	6a	Clearly define the outcome that is predicted by the prediction model, including how and when assessed.	6-9
	6b	Report any actions to blind assessment of the outcome to be predicted.	n/a
Predictors	7a	Clearly define all predictors used in developing or validating the multivariable prediction model, including how and when they were measured.	6-9
	7b	Report any actions to blind assessment of predictors for the outcome and other predictors.	n/a
Sample size	8	Explain how the study size was arrived at.	5
Missing data	9	Describe how missing data were handled (e.g., complete-case analysis, single imputation, multiple imputation) with details of any imputation method.	10
Statistical analysis methods	10a	Describe how predictors were handled in the analyses.	10
	10b	Specify type of model, all model-building procedures (including any predictor selection), and method for internal validation.	10
	10d	Specify all measures used to assess model performance and, if relevant, to compare multiple models.	n/a
Risk groups	11	Provide details on how risk groups were created, if done.	n/a
**Results**			
Participants	13a	Describe the flow of participants through the study, including the number of participants with and without the outcome and, if applicable, a summary of the follow-up time. A diagram may be helpful.	n/a
	13b	Describe the characteristics of the participants (basic demographics, clinical features, available predictors), including the number of participants with missing data for predictors and outcome.	n/a
Model development	14a	Specify the number of participants and outcome events in each analysis.	n/a
	14b	If done, report the unadjusted association between each candidate predictor and outcome.	n/a
Model specification	15a	Present the full prediction model to allow predictions for individuals (i.e., all regression coefficients, and model intercept or baseline survival at a given time point).	n/a
	15b	Explain how to the use the prediction model.	n/a
Model performance	16	Report performance measures (with CIs) for the prediction model.	n/a
**Discussion**			
Limitations	18	Discuss any limitations of the study (such as nonrepresentative sample, few events per predictor, missing data).	n/a
Interpretation	19b	Give an overall interpretation of the results, considering objectives, limitations, and results from similar studies, and other relevant evidence.	n/a
Implications	20	Discuss the potential clinical use of the model and implications for future research.	11
**Other information**			
Supplementary information	21	Provide information about the availability of supplementary resources, such as study protocol, Web calculator, and data sets.	n/a
Funding	22	Give the source of funding and the role of the funders for the present study.	11

We recommend using the TRIPOD Checklist in conjunction with the TRIPOD Explanation and Elaboration document.

For peer review only
